# Geographic population structure and distinct intra-population dynamics of globally abundant freshwater bacteria

**DOI:** 10.1093/ismejo/wrae113

**Published:** 2024-07-03

**Authors:** Matthias Hoetzinger, Martin W Hahn, Linnéa Y Andersson, Nathaniel Buckley, Chelsea Ramsin, Moritz Buck, Julia K Nuy, Sarahi L Garcia, Fernando Puente-Sánchez, Stefan Bertilsson

**Affiliations:** Department of Aquatic Sciences and Assessment, Swedish University of Agricultural Sciences, 750 07 Uppsala, Sweden; Faculty of Chemistry, Biotechnology and Food Science, Norwegian University of Life Sciences, 1433 Ås, Norway; Research Department for Limnology, University of Innsbruck, 5310 Mondsee, Austria; Department of Aquatic Sciences and Assessment, Swedish University of Agricultural Sciences, 750 07 Uppsala, Sweden; Department of Aquatic Sciences and Assessment, Swedish University of Agricultural Sciences, 750 07 Uppsala, Sweden; Department of Aquatic Sciences and Assessment, Swedish University of Agricultural Sciences, 750 07 Uppsala, Sweden; Department of Aquatic Sciences and Assessment, Swedish University of Agricultural Sciences, 750 07 Uppsala, Sweden; Department of Ecology, Environment, and Plant Sciences, Science for Life Laboratory, Stockholm University, 104 05 Stockholm, Sweden; Centre for Water and Environmental Research, University of Duisburg-Essen, 45141 Essen, Germany; Department of Ecology, Environment, and Plant Sciences, Science for Life Laboratory, Stockholm University, 104 05 Stockholm, Sweden; Institute for Chemistry and Biology of the Marine Environment (ICBM), Carl von Ossietzky Universität Oldenburg, 26129 Oldenburg, Germany; Department of Aquatic Sciences and Assessment, Swedish University of Agricultural Sciences, 750 07 Uppsala, Sweden; Department of Aquatic Sciences and Assessment, Swedish University of Agricultural Sciences, 750 07 Uppsala, Sweden

**Keywords:** population structure, freshwater bacteria, population genomics, metagenomics, microevolution, speciation

## Abstract

Implications of geographic separation and temporal dynamics on the evolution of free-living bacterial species are widely unclear. However, the vast amount of metagenome sequencing data generated during the last decades from various habitats around the world provides an unprecedented opportunity for such investigations. Here, we exploited publicly available and new freshwater metagenomes in combination with the genomes of abundant freshwater bacteria to reveal geographic and temporal population structure. We focused on species that were detected across broad geographic ranges at high enough sequence coverage for meaningful population genomic analyses, associated with the predominant freshwater taxa acI, LD12, *Polynucleobacter*, and *Candidatus* Methylopumilus. Despite the broad geographic ranges, each species appeared as a sequence-discrete cluster, in contrast to abundant marine taxa, for which continuous diversity structures were reported on a global scale. Population differentiation increased significantly with spatial distance in all species, but notable dispersal barriers (e.g. oceanic) were not apparent. Yet, the different species showed contrasting rates of geographic divergence and strikingly different intra-population dynamics in time series within individual habitats. The change in an LD12 population over 7 years was minor (*F*_ST_ = 0.04) compared to differentiation between lakes, whereas a *Polynucleobacter* population displayed strong changes within merely 2 months (*F*_ST_ up to 0.54), similar in scale to differentiation between populations separated by thousands of kilometers. The slowly and steadily evolving LD12 population showed high strain diversity, whereas the dynamic *Polynucleobacter* population exhibited alternating clonal expansions of mostly two strains only. Based on the contrasting population structures, we propose distinct models of speciation.

## Introduction

Evolution requires populations to diverge, eventually far enough to form different species. The importance of geographic separation in this process has long been recognized: “...barriers of any kind, or obstacles to free migration, are related in a close and important manner to the differences between the productions of various regions.” [1]. However, in comparison to most macroorganisms, bacteria are exceedingly mobile due to their minuscule size, which facilitates long-range dispersal, for instance, via clouds [2–5], ocean currents [6–8], or by hitchhiking with larger migratory organisms [9–11]. Examples of entire orders being restricted to certain continents, as is the case for several mammals [12], are thus not to be expected for bacteria. Still, geographic endemism occurs for microbes, albeit at a higher phylogenetic resolution. A recent study relating average nucleotide identities (ANI) between publicly available genomes to geographic distances between their sites of origin provided valuable insights into the phylogenetic scales at which prokaryotes from different environments display endemism [13]. On one end of the spectrum, prokaryotes from the subsurface showed the highest level of endemism. No clades above the commonly used species delineation threshold of 95% ANI [14, 15] were found on opposite hemispheres of the earth. On the other end, for marine environments, clades were not restricted to a single hemisphere, unless defined with an ANI threshold of 99.9997% or greater. Hence, even marine bacterial strains (if defined with a 99.5% ANI threshold as in [16]), are likely to be globally distributed. Lake prokaryotes showed an intermediate level of endemism with a predicted probability of being found on opposite hemispheres approaching zero for clades >99.6% ANI [13], which implies that analyses of lake bacteria on species level would exhibit little geographic signal, whereas intra-species divergence may still be affected by dispersal limitation. This would open the possibility for local adaptation and ultimately enable allopatric speciation. Yet, the impact of geographic separation on intra-species divergence of freshwater bacteria has hardly been studied so far [17]. To address this knowledge gap, we quantified allopatric divergence in several freshwater bacterial species on continental and inter-continental scales. To gauge its relevance for evolution, it is important to consider its magnitude relative to the variation happening within habitats over contemporary timescales. Although temporal dynamics within bacterial communities (community or population dynamics) are well described in certain freshwater habitats [18, 19], only a few studies have focused on variation within populations over time (intra-population dynamics) [20, 21], limiting our understanding of microevolutionary processes. To that end, we also unravel the temporal dynamics of intra-species nucleotide diversity. Genome-wide data is necessary to resolve the phylogenetic scales relevant to this study. Molecular clocks suggest that bacterial intra-species divergence happens in a range of up to a few million years. For instance, 99% ANI may correspond to 1000–1 500 000 years of divergence [22–24], whereas 99% 16S rRNA gene identity was estimated to typically correspond to more than 10 million years divergence time [25]. The 16S rRNA gene is thus of limited value for reconstructing intra-species divergence. However, conspecific freshwater bacterial genomes are scarce [13] and therefore also population genomic insights. To overcome this data paucity, we leveraged publicly available as well as newly sequenced freshwater metagenomes from around the world. Through read mapping against a previously selected set of reference genomes and analysis of nucleotide polymorphisms in the metagenomes, we aimed to assess: (i) the genomic coherence of freshwater bacterial species over global scales, (ii) the impact of geographic separation on population divergence, and (iii) the temporal dynamics of intra-population diversity. We thereby revealed insights into population structures and differences among species, which may contribute to a better understanding of bacterial evolution.

## Materials and methods

### Reference genomes

The 20 reference genomes, 18 from isolated strains and 2 SAGs, were characterized by ANI <95% for all pairwise comparisons (Supplementary Table S1). They were chosen based on several publications that detected the respective species in different habitats with high relative abundance or across a broad geographic range [17, 26–31]. They were associated with five different genera (*Candidatus* Fonsibacter, *Candidatus* Methylopumilus, *Candidatus* Nanopelagicus, *Candidatus* Planktophila, and *Polynucleobacter*). The selection of reference genomes was biased by the personal preference of the first author (e.g. 13 of the 20 were *Polynucleobacter* genomes). The seven finally analyzed species should thus not be regarded as a fair representation of the most abundant or prevalent bacteria in global freshwater ecosystems but as a rather arbitrary yet widespread and abundant subset of those.

### Metagenomes

NCBI SRA was screened for aquatic metagenomes by querying Illumina whole genome sequenced datasets with any of the keywords: “water metagenome,” “peat metagenome,” “seawater metagenome,” “marine metagenome,” “marine plankton metagenome,” “marine sediment metagenome,” “karst metagenome,” “lagoon metagenome,” “lake water metagenome,” “glacier lake metagenome,” “freshwater metagenome,” “freshwater sediment metagenome,” “aquatic metagenome,” “bog metagenome,” “drinking water metagenome,” “estuary metagenome,” “Winogradsky column metagenome,” “alkali sediment metagenome,” “sediment metagenome,” or “ground water metagenome.” We used a custom script (filtering_and_converting_coordinates.py) to filter this table for lentic freshwater metagenomes with associated GPS coordinates. The script used keywords to exclude (e.g. sediment, viral, and river) and filtered for the string “fresh” in the metagenome metadata, and further checked for entries in various columns containing geographic coordinates. The initial table of 21 806 metagenomes was thereby reduced to 1646 metagenomes. The aim was not to screen public metagenomes exhaustively, and our script likely removed metagenomes that were sampled from lentic freshwater habitats and for which GPS coordinates could have been obtained by more elaborate screening. We complemented these 1646 metagenomes with the 273 metagenomes of the stratfreshDB [32]. Besides those previously published metagenomes, we generated 33 metagenomes (Supplementary Table S2) for this study, amounting to 1952 lentic freshwater metagenomes used to screen for the target species. The newly generated metagenomes were obtained from a *Polynucleobacter paneuropaeus* (*P. pan.*)-specific selection of humic lakes and ponds in the Austrian Alps and Northern Lapland. The selection was based on high observed relative abundances of *P. pan.* in an amplicon dataset of a protein-encoding gene [30] and detection based on isolated genomes [17]. Fourteen metagenomes were sampled in 2018 and nineteen in 2020, and a total of 20 are part of a time series from Pond EnzMain in the Austrian Alps. The 33 samples (400–700 ml each) were sequentially vacuum filtered through 0.8 and 0.2 μm Whatman Nucleopore filters (47 mm diameter) to enrich for the relatively small *P. pan.* bacteria [33] on the latter filters. For both 0.8 and 0.2 μm filtration, two to four filters were used for each sample, as they typically clogged after passing through ~150 ml of the humic waters. DNA was extracted from only the 0.2 μm filters for the 2018 samples, but from both 0.8 and 0.2 μm filters for the 2020 samples. DNA was extracted using phenol-chloroform-isoamyl alcohol as previously described [34]. The 0.8 and 0.2 μm fractions of the metagenomes from 2020 were sequenced separately and have different SRA accessions in NCBI but were combined for the analyses in this paper to obtain higher coverage, as *P. pan.* was also detected in the 0.8 μm fractions. Details about the samples, library preparation, and sequencing methods are given in Supplementary Table S2.

### Preselection of metagenomes/species

To avoid a computationally intensive mapping of all 1952 metagenomes against all 20 reference genomes, we first screened the metagenomes for the presence of our five target genera using the taxonomic classification of assembled MAGs. Here, all public metagenomes were assembled using MEGAHIT v1.2.9 [35] with “—min-contig-len” set to 2500, after downloading from SRA with parallel-fastq-dump (https://github.com/rvalieris/parallel-fastq-dump) and quality filtering with fastp v0.20.1 [36]. MAGs were binned with MetaBAT 2 v2.15 [37] using a coverage data file with the “-a” flag and the minimum bin size set to 500 000 with the “-s” flag. The coverage data file was generated before with the “jgi_summarize_bam_contig_depths” command implemented in MetaBAT 2 using a sorted bam file obtained by mapping reads with Bowtie 2 v2.4.4 [38] and running “samtools view” and “samtools sort” from SAMtools v1.10 [39] on the sam output file from Bowtie 2. Scripts used for the whole workflow can be found at (https://github.com/moritzbuck/SRAnsack). Completeness and contamination of the MAGs were estimated with CheckM v1.2.0 [40] based on universal prokaryotic single marker genes using “taxonomy_wf life Prokaryote.” MAGs >30% complete and <3% contaminated were taxonomically classified using GTDB-Tk v2.1 [41] with the GTDB release 207 [42]. The MAGs of each metagenome were finally screened for the presence of the target genera using the GTDB-Tk classification, to narrow down the initial set of metagenomes to be mapped against each reference genome. The MAGs were not used for mapping or any other analysis in this study and thus had no further influence on the results.

To further prefilter the obtained set of metagenomes on a species level, a fraction of each metagenome was mapped against the reference genomes. To this end, the first 10% of reads from the fastq files were downloaded using fastq-dump from the SRA Toolkit [43] and mapped against the reference genomes using Bowtie 2 [38] with a 95% identity cutoff (settings: --ignore-quals --mp 1,1 --rdg 0,1 --rfg 0,1 --score-min L,0,-0.05). Average coverage depth across the reference genome was computed using SAMtools [39], BEDTools [44], and the “gen_contig_cov_per_bam_table.py” script from the MetAssemble software package (https://github.com/inodb/metassemble) in a slightly modified form (https://github.com/thr44pw00d/population-structure). Metagenome/species pairs with an average coverage depth of the reference genome of ≥2 (corresponding to ~20 in the complete metagenome) and a coverage breadth of ≥50% were considered potentially useful for population genomic analysis. Only those species that showed a broad geographic range when applying these criteria were used in further analyses. The respective metagenomes were downloaded completely and used for the population genomic analyses described below.

### Population genomics

We used POGENOM v0.8.3 [45] to determine allele frequencies in the metagenomes and calculate π and *F*_ST_ values. To generate the therefore necessary vcf files, the “input_pogenom.sh” pipeline was run separately for each species, mapping the pre-selected metagenomes against the reference genome. Parameters were set to realize ≥95% identity for the Bowtie2 mapping, a median coverage depth ≥30 and a coverage breadth ≥50% at a mapping quality ≥20 for metagenomes to be included. Metagenome/species pairs that fulfilled these criteria are given in Supplementary Table S3, and population genomic results are in Supplementary Table S4. Mapped reads were subsampled to obtain a median genome-wide coverage depth of 30 for each metagenome to omit biases from unequal coverage in different metagenomes. Furthermore, we ran POGENOM with the following tweaks to calculate π based on the actual rather than an estimated number of included loci (newer versions of POGENOM may do so by default). Adding the “--report-monomorphic” flag to the “freebayes_parameters” and” QUAL > NA” as settings for “vcffilter_qual” in the input POGENOM config file ensured that the pipeline generated a vcf file that contained all loci (including monomorphic loci and without filtering on the probability of loci being polymorphic). The vcf file was subsequently filtered using a custom Python script to keep only loci with >99% estimated probability of being mono- or polymorphic, respectively. The “pogenom.pl” script was then run twice, first with the “--pi_only” flag. This run was just to get the number of analyzed loci. All other results were obtained from the second run, where the “--genome_size” was set to the number of loci determined in the first run. In both runs, the filtered vcf file was used, with “--min_count” set to 15 and “--min_found” to the number of metagenomes. This means that only loci covered at least 15-fold in each metagenome were included. Thus, analyses results refer to the core genome of each species. The coverage at each included locus was finally subsampled to 15 using the “--sumbsample” flag to ensure that each locus was represented by the same number of observations. Obtained population genomic measures should thus be well comparable between the different metagenomes and species. Linkage disequilibrium was computed from the merged bam files obtained from POGENOM using InStrain v1.6.3 [46].

### BLAST read mapping

For read mapping to reference genomes using BLAST, each metagenome was subsampled to 1 million reads using seqtk v1.2-r101 (https://github.com/lh3/seqtk). Fastq files were transformed to fasta using Fastx v0.0.14 (https://github.com/agordon/fastx_toolkit), whereby reads containing unknown nucleotides (Ns) were discarded. Read mapping was done as previously described in [47] (settings: -task “blastn” -evalue 0.01 -max_target_seqs 10 -perc_identity 70). BLAST results were filtered for a minimum read length of 70 bp and an alignment length ≥90% of the read length using the TabularBlast_ShortRead_Filter.py script (https://github.com/rotheconrad/GoM). Histograms of the percent identities of the filtered hits were plotted using the hist function in R v4.2.2 [48].

### Exclusion of metagenomes

To obtain the final set of metagenomes used for each species, metagenomes that fulfilled the POGENOM criteria were filtered to exclude those for which a substantial amount of the reads mapped at ≥95% identity may have originated from a sister species. This was done by removing metagenome/species pairs for which the number of blast hits with 90%–95% identity was higher than the number of hits with 95%–100% identity. Blast histograms of the final set of metagenome/species pairs are shown in Supplementary Fig. S1 and of the excluded ones in Supplementary Fig. S2, concerning a total of eight metagenomes associated with F. sp., F. ubi., P. ver., and M. uni.

### Calculation of intra-species diversity

ANIr_95_ was calculated from the blast mapping as the average percent identity of reads that mapped with ≥95% identity to the reference genome and is thus a measure of similarity between the resident population and the reference genome. The influence of the choice of the reference genome on ANIr_95_ is illustrated in Supplementary Fig. S3, which shows that ANIr_95_ values tend to be higher in the reference genomes’ home habitats. For computing species-wide ANIr_95_ and the blast histograms, the percent identity values of the mapped reads from the different metagenomes used for each species were combined. These values and the breadth of the distribution in the histograms provide insights into the coherence of the species as represented in the analyzed metagenome sets.

The other used intra-population diversity measures (π, proportion of polymorphic loci, and *H*_noncorr_) were computed from the POGENOM output. As these are based on polymorphisms within the mapped reads, they are not influenced by the choice of reference genome. Theoretically, the loci considered for calculating these measures could change depending on the reference genome used, but as we only included loci that were sufficiently covered in all metagenomes, the analyses refer to “core loci” expected to be present in almost all genomes of the species, whereas the more flexible part of the genomes would have been excluded. The nucleotide diversity π was obtained directly from the “intradiv.txt” file in the POGENOM output. The proportion of polymorphic loci was calculated as “number of polymorphic loci” * 100/“number of analyzed loci”, whereby the number of polymorphic loci in each metagenome was obtained from the “allele-freqs.txt” file that was output by POGENOM. Estimates of strain diversity were computed from the “allele-freqs.txt” file as the sum of the Shannon entropies of the allelic frequencies of all the non-correlated polymorphic loci (*H*_noncorr_) according to the following rationale.

(1) Let L_1_ be a polymorphic locus of frequencies [f_i_,f_j_,f_k_,f_l_], sorted from highest to lowest.
  - E.g. L_1_ = [f_A_ = 0.6, f_C_ = 0.3, f_T_ = 0.2, f_G_ = 0.1].
(2) The frequencies of L_1_ in the population can be explained by the presence of four strains with the same frequencies as L_1_ [f_i_,f_j_,f_k_,f_i_], each bearing a different allele in L_1_.
  - E.g. S_1_^L1:A^ = 0.6, S_2_^L1:C^ = 0.3, S_3_^L1:T^ = 0.2, S_4_^L1:G^ = 0.1).
(3) The Shannon strain diversity for that species in that population would thus be *H*(f_i_,f_j_,f_k_,f_l_), where *H* is the Shannon entropy of a vector of frequencies.
  - E.g. *H*(L_1_) = *H*(0.6, 0.3, 0.2, 0.1) = 1.20
(4) Let L_2_ be a second polymorphic locus whose sorted allelic frequencies are similar or highly correlated to those of L_1_.
  - E.g. L_2_ = [f_T_ = 0.6, f_A_ = 0.3, f_C_ = 0.2, f_G_ = 0.1]).
(5) The frequencies of L_1_ and L_2_ in the population can be parsimoniously explained by the same number of strains than the frequencies of L1 alone.
  - E.g. S_1_^L1:A;L2:T^ = 0.6, S_2_^L1:C;L2:A^ = 0.3, S_3_^L1:T;L2:G^ = 0.2, S_4_^L1:G;L2:G^ = 0.1).
(6) Observing several loci with highly correlated sorted allelic frequencies should thus not result in an increased estimated strain diversity.
  - E.g. *H*_noncorr_(L_1_;L_2_) = *H*_noncorr_(L_1_)
(7) Let L_3_ be a third polymorphic locus whose sorted allelic frequencies [f_i’_,f_j’_,f_k’_,f_l’_] are not correlated to those of L_1_ and L_2_.
  - E.g. L_3_ = [f_G_ = 0.4, f_T_ = 0.4, f_A_ = 0.2, f_C_ = 0].
(8) Two loci with non-correlated sorted allelic frequencies can in most cases not be explained by the same number of strains as a single locus, and thus should result in an increased estimated strain diversity.
  - E.g. *H*_noncorr_(L_1_;L_2;_L_3_) = *H*_noncorr_(L_1;_L_2_) + *H*(L_3_) = 1.20 +  1.05 = 2.25

This is a pragmatic approach meant to provide a fast approximation to intra-species strain diversity without the need for haplotype reconstruction. We benchmarked it using a synthetic dataset, showing that it provides a more accurate representation of strain diversity than π (Supplementary Fig. S4, Supplementary Table S5), which will always increase with the number of polymorphic loci. In this work, our threshold for considering two loci as correlated was 1, meaning that only loci with identical sorted allelic frequencies were collapsed, but lower thresholds may be desirable to accommodate noise and sequencing errors, particularly if the coverage depth per locus is higher than the one used in this study.

To compare diversity measures between species, we tested for normality using the Shapiro–Wilk test (“shapiro.test”) and for homogeneity of variances using the Bartlett test (“bartlett.test”) in R v4.2.2. For none of the diversity measures, all species showed a normal distribution or equal variances. Differences between species were thus tested using the non-parametric Wilcoxon-Mann–Whitney Rank Sum test (“wilcox.test”) in R v4.2.2.

### Divergence with spatial distance and time

Analyses were done in R v4.2.2. Spatial distances were calculated using the distm function (fun = distGeo) from the geosphere package. Time differences were calculated using the difftime function. To assess correlations of F_ST_ values with spatial distances and time differences, Mantel tests were conducted using the Mantel function (method = “spearman”, permutations = 10 000) from the vegan package. Linear regressions were computed using the stat_smooth function (method = “lm”) from the ggplot2 package. Weighted principal coordinate analyses of F_ST_ values were computed using the wcmdscale function (*k* = 2, eig = TRUE) from the vegan package. Ellipses edging continents and habitats were drawn using the ordiellipse function (scaling = “symmetric”, kind = “ehull”).

### Intra-population dynamics

Allele frequency spectra for the different metagenomes, giving the proportion of each base at each polymorphic locus based on 15-fold coverage, were obtained from the POGENOM results. For clustering alleles in the *P. pan.* and F. sp. time series into subpopulations or putative strains, Spearman rank correlations between alleles were calculated based on allele frequencies over time using the spearmanr function from the scipy.stats module in Python. All the following analyses were done in R. Correlation distances were calculated as 1 − r and visualized in a matrix using the pheatmap function. Correlation distances were clustered using hclust. To infer the number of groups, the as.clustrange function from the WeightedCluster package was run for up to 20 groups (ncluster = 20), and the number of groups was chosen based on PBC (Point Biserial Correlation). The hierarchically clustered alleles were split into the respective number of groups using the cutree function, and the split was visualized on the tree using the dendextend package. The dynamics of the groups across the time series were plotted using ggplot2, after calculating the mean allele frequency of all alleles in each group for each metagenome.

### Definition of terms that are frequently used with different meanings elsewhere

Allele: nucleotide variant at a given locus.

Habitat: Lake, pond, or other freshwater sampling site.

Locus: a single nucleotide position in the genome.

Polymorphism: a locus with two or more alleles.

Population: conspecific organisms co-occurring in a habitat.

Species: group of organisms sharing >95% ANI (genomospecies).

## Results and discussion

### Abundance and distribution of the investigated species

A set of 1952 lentic freshwater metagenomes was used to screen for 20 initially selected reference genomes, representing 20 different genomospecies (<95% ANI for all pairs) that were previously observed to be abundant in freshwater datasets [17, 26–31]. They are associated with the five genera *Ca.* Fonsibacter (*Alphaproteobacteria*, LD12 group), *Ca.* Methylopumilus (*Betaproteobacteria*), *Ca.* Nanopelagicus (*Actinomycetota*, acI group), *Ca.* Planktophila (*Actinomycetota*, acI group), and *Polynucleobacter* (*Betaproteobacteria*). Seven of the species were detected with coverage exceeding our thresholds (genome-wide median coverage depth ≥30 at ≥95% sequence identity and ≥20 mapping quality) at a broad geographic range (spanning at least 2500 km) to allow for meaningful population genomic analyses at continental and even inter-continental scales (Table 1, Supplementary Fig. S5). The finally analyzed dataset contained 225 metagenomes, with 10–111 metagenomes per species (Supplementary Table S3). Relative abundances assessed as percentage of metagenome bases mapped to a reference genome (this measure differs from relative cellular abundance as it depends on genome size and genome copy numbers per cell) were highest for *P. pan.* in several humic ponds, with up to 13.2% mapped bases in an Alpine pond (Supplementary Table S3, Supplementary Figs S5 and S6). Similarly, *Polynucleobacter finlandensis* (*P. fin.*) recruited up to 11.6% of bases from the metagenomes of the humic Trout Bog Lake. The two species of the LD12 clade were detected at a maximum relative abundance of 6.1% in Lake Biwa (*Ca.* Fonsibacter ubiquis (F. ubi.)) and 5.4% in Lake Michigan (*Ca.* Fonsibacter sp. (F. sp.)). The two acI species recruited up to 1.7% in Lake Michigan (*Ca.* Nanopelagicus abundans (N. abu.)) and 0.9% in Lake Loclat (*Ca.* Planktophila vernalis (P. ver.)), and *Ca.* Methylopumilus universalis (M. uni.) accounted for up to 0.7% in the Římov reservoir. However, sampling procedures (e.g. prefiltration) and sequencing methods varied for the different metagenomes, and relative abundances are thus not directly comparable among metagenomes. It was still obvious that the two *Polynucleobacter* species thrived in acidic waters rich in humic matter, and they were also found to be abundant and coexisting in a few small habitats in Fennoscandia (Supplementary Table S3). In contrast, the other five species thrived in mostly larger lakes with neutral to slightly alkaline pH, where the same type of “abundant coexistence” was apparent in several lakes, with up to four of the species coexisting in Lake Zurich. Although *P. pan.* and P. ver. were not detected outside Europe, at least not with high enough coverage to enable population structure to be described, the five other species covered multiple continents. F. ubi. spanned the largest geographic range of 16 200 km, from an urban water sample in Singapore to Lake Eufaula in Georgia, USA.

**Table 1:** The seven investigated species and the respective reference genomes used for mapping of metagenomes.

**Species**	**Abbreviation**	**Reference genome**	**References**	**Reference genome source**	**Reference genome NCBI accession**	**Reference genome size (bp)**	**Metagenomes** ^**a**^	**Habitats** ^**b**^	**Analyzed loci** ^**c**^	**Polymorphic loci** ^**d**^
*Ca.* Fonsibacter sp.	F. sp.	AAA028-D10 (SAG)	[27]	Lake Mendota	GCA_000510845.1	925 141	23	7	296 334	7459
*Ca.* Fonsibacter ubiquis	F. ubi.	LSUCC0530	[26]	Lake Borgne	GCF_002688585.1	1 160 202	19	11	384 503	6242
*Ca.* Methylopumilus universalis	M. uni.	MMS-RIV-30	[29]	Rimov reservoir	GCF_006364215.1	1 268 083	38	9	539 371	10 365
*Ca.* Nanopelagicus abundans	N. abu.	MMS-IIB-91	[28]	Lake Zurich	GCF_002288305.1	1 161 863	10	3	508 148	11 124
*Ca.* Planktophila vernalis	P. ver.	MMS-IIA-15	[28]	Lake Zurich	GCF_002288185.1	1 364 004	13	5	430 975	13 209
*Polynucleobacter finlandensis*	*P. fin.*	MWH-Mekk-B1	[31]	Lake Mekkojärvi	GCF_018881755.1	2 280 072	111	14	905 581	19 467
*Polynucleobacter paneuropaeus*	*P. pan.*	UB-Kaiv-W7	[33]	Pond Kaivoslampi	GCF_003261255.1	1 830 921	37	14	1 106 814	15 830

a Number of metagenomes that passed the coverage thresholds for the respective reference genome and were finally analyzed.

b Number of different habitats associated to the analyzed metagenomes.

c Number of loci that passed the coverage thresholds and were analyzed in each metagenome used for the species.

d Number of loci that were polymorphic in at least one of the metagenomes used for the species.

### Species demarcation

**Figure 1:**
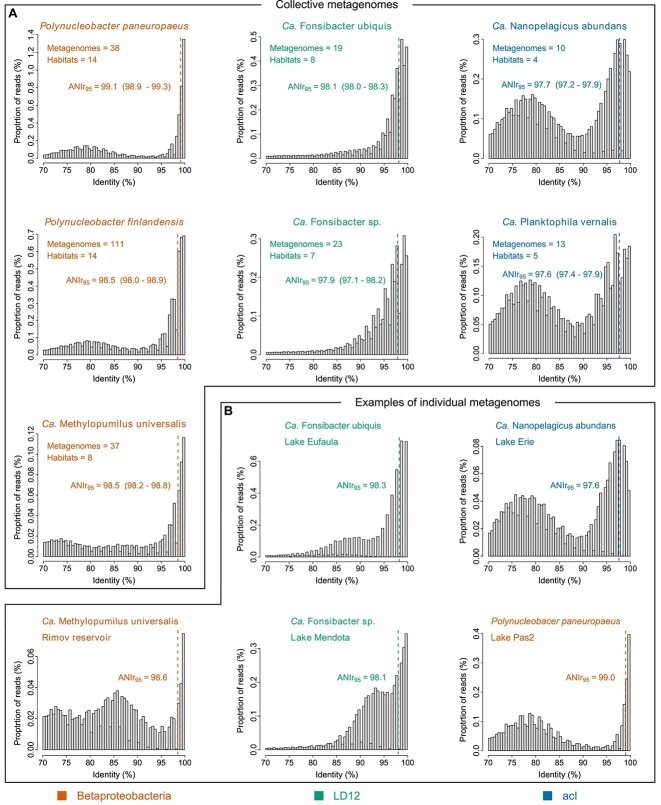
Histograms of metagenome reads mapped to the reference genomes by BLAST: (A) Collective mappings of metagenomes from different locations. The numbers of metagenomes and habitats underlying each plot are given on the upper left. For each species the ANIr_95_ value across all respective metagenomes is given and indicated by a dashed line. The range of ANIr_95_ values when computed for the respective metagenomes separately is given in brackets. (B) Selected examples of individually mapped metagenomes. All histograms from individual metagenomes are shown in Supplementary Fig. S1.

As we define species based on DNA sequence identity in this study, we first need to discuss the feasibility and relevance of such an approach. It has been shown that bacterial genomes of isolates as well as metagenome-assembled genomes (MAGs) have a striking sparsity of ANIs between 83 and 96%. This gap has been used to operationally define species based on 95% ANI [14, 15]. However, the relevance of this threshold as a universal genomic boundary is a matter of debate and has been questioned as a potential artefact from biased isolation of strains or assembly of MAGs [49, 50]. Such arguments are rebutted by the detection of sequence-discrete populations, discernable species separated by genetic discontinuity observed in natural communities, using metagenomic read mapping [20, 47, 51–53]. So far, sequence-discrete populations were observed locally, within certain habitats, and mapping of metagenomes from distant environments against marine prokaryotic reference genomes suggested a more continuous diversity structure at global scales [51, 54]. In contrast, here we observed sequence-discrete species over continental as well as global scales by collective mapping of reads from geographically distant habitats (Fig. 1A). It remains to be shown if such a discontinuous diversity structure is generally more common in freshwater than marine bacteria.

Although all species showed distinct peaks of mapped reads at >95% identity, diversity within species differed considerably. The ANIr_95_ (ANI of the metagenome reads mapped to the reference genome with ≥95% identity) values from the collectively mapped metagenomes (Fig. 1A) were highest for *Polynucleobacter* (99.1% for *P. pan*.), suggesting low intra-species diversity, whereas they were notably lower for acI (97.6% for P. ver.), suggesting high diversity. This aligns well with previous observations of genome comparisons of isolated strains that hardly showed conspecific strain**-**pairs with <97% ANI for *Polynucleobacter* [31, 55], whereas strain-pairs with ANI values around 95% were more common for acI [28]. Besides differences in intra-species diversities, the taxa also differed in the clarity of the “species gap” (sparsity of reads mapping between 85% and 95% identity). In blast histograms from individual metagenomes (Fig. 1B), peaks of mapped reads between 85% and 95% identity were occurring in LD12, indicating co-occurring closely related species. In line with that, previous comparisons of LD12 single-amplified genomes (SAGs) from different lakes frequently showed ANIs between 85% and 95% [53]. This suggests that diversity structure tends to be more continuous in LD12, somewhat reminiscent of its marine sister clade SAR11 [54]. In M. uni., peaks of mapped reads from putatively related species frequently appeared around 85% identity, whereas such peaks appeared almost exclusively at ≤80% identity for *Polynucleobacter* and acI, suggesting clearer species boundaries for the latter taxa (see Supplementary Fig. S1 for all blast histograms).

Overall, the identity distributions of mapped metagenome reads corroborate the usefulness of the commonly used 95% ANI threshold for bacterial species delineation, given the peaks in the histograms at >95% identity. Nevertheless, intra-species diversity as well as the frequency of putative sister species occurring within the species gap varied considerably among taxa. Hence, the 95% ANI threshold is not equally suited for delineating all different taxa. It should rather be seen as a pragmatic (lower) limit for species delineation that works well for many taxa, but not as a token for a universal composition of intra- and inter-species divergence.

### Intra-population diversity

**Figure 2:**
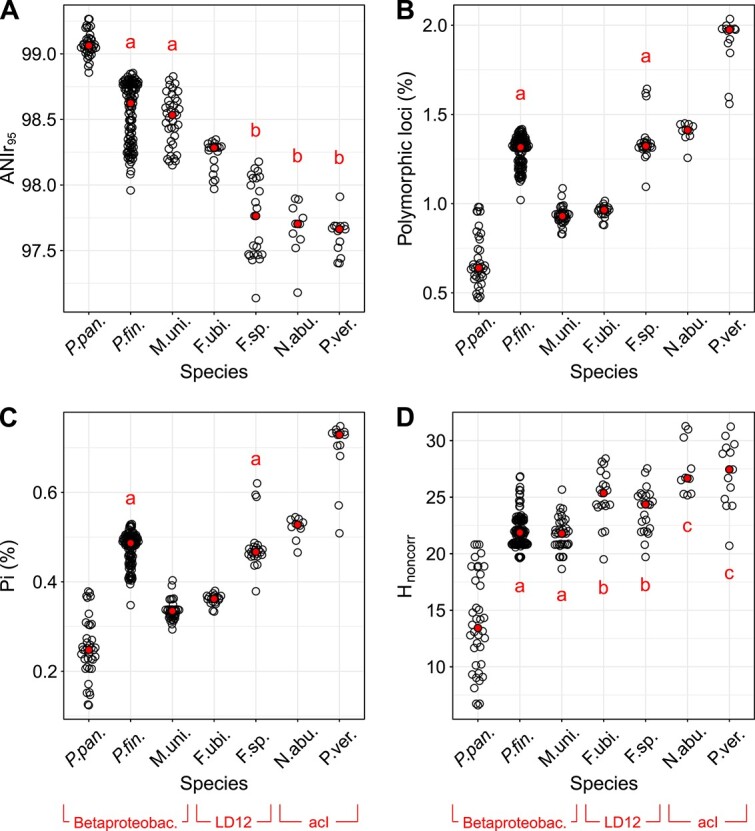
Intra-population diversities for the seven species according to different metrics. Each dot refers to one metagenome. Median values are shown as filled red dots. Pairs that are not significantly different (*P* > .05) according to Wilcoxon-Mann–Whitney rank sum test are marked with a common letter.

To characterize intra-population diversity beyond ANIr_95_ (Fig. 2A), we calculated other measures of genomic variation in metagenomes based on mappings with ≥95% identity thresholds (Supplementary Table S4). A simple measure giving the proportion of polymorphic loci (Fig. 2B) showed very similar patterns to the nucleotide diversity (π), which can be interpreted as the average dissimilarity between two randomly picked individuals of the same population (Fig. 2C). The proportion of polymorphic loci ranged from 0.47% (=4700 SNPs per Mbp of genome) in *P. pan.* to 2.04% in P. ver., and π from 0.12% to 0.75%. Besides these diversity measures, it would be interesting to know the strain diversity, especially for analyzing intra-population dynamics (see below). This would help to clarify if the abundance dynamics of different strains are uncoupled, for instance, due to environment-dependent fitness differences that may also include strain-specific differences in predation susceptibility. Strain diversity is expected to correlate with nucleotide diversity, yet a given nucleotide diversity could be realized by a higher number of closely related strains or by a lower number of distantly related strains. To calculate a diversity metric that represents strain diversity better than π, we used Shannon entropies of polymorphic loci with non-correlated allele frequencies (*H*_noncorr_). We benchmarked our method to compute *H*_noncorr_ as a proxy for strain diversity on a simplified dataset, which showed that it reflected strain diversity better than π (Supplementary Fig. S4, Supplementary Table S5). In the natural data, it roughly divided the seven species into four groups of increasing diversity (*P. pan.*, *P. fin.* + M. uni., F. ubi. + F. sp., and N. abu. + P. ver.), whereby *P. pan.* showed by far the lowest median *H*_noncorr_ (Fig. 2D). Comparing π and *H*_noncorr_ provides hints about intra-population structure. For instance, H_noncorr_ suggests that *P. fin.* and M. uni. populations tend to comprise similar numbers of strains, whereas π is significantly higher in *P. fin*. We may conclude that ANI between co-existing strains tends to be lower in *P. fin.* compared to M. uni. This study covers allelic diversities within the core genome, although intra-species diversity in prokaryotes can to a large extent stem from gene content diversity, i.e. the accessory genome. The implications of the accessory genome on the evolution of prokaryotic species are certainly substantial, yet, beyond the scope and reach of this study.

### Homologous recombination

Linkages between nearby alleles in the genome give hints about homologous recombination rates, although other factors, such as selection, may influence these linkages too. Loci are in linkage disequilibrium [56] when the association of their allele frequencies is non-random. Nearby loci are more likely to be transferred together in recombination events and are thus expected to show linkage disequilibrium in recombinogenic bacteria. Linkage measured as the normalized squared correlation coefficient *R*^2^_norm_ was highest in *P. pan.*, up to 0.7 between neighboring alleles (Supplementary Fig. S7). A maximum value of 1 would mean that pairs of alleles at polymorphic loci with a given distance always occur together on the same read or read pair. Linkage approached a baseline around 350 bp, suggesting that the sequence length of most homologous recombination events was shorter than that (cf. [57]). Homologous recombination in the genomes of isolated *P. pan.* strains inferred with a model based on coalescent theory also suggested a short average recombination tract length of 66 bp [17]. For comparison, the median recombination tract length inferred for *Bacillus cereus* using a similar method was 236 bp [58]. The other six species showed lower linkage disequilibrium than *P. pan.*, with maximum *R*^2^_norm_ values ranging from 0.3 (F. sp.) to 0.5 (*P. fin.*) and *R*^2^_norm_ versus distance plateauing earlier, between 100 bp (N. abu.) and 300 bp (*P. fin.*). High homologous recombination rates in *P. pan.* were suggested earlier to counteract divergence [17] and might explain its low intra-species diversity. For the other species, a clear relationship between intra-species diversity (Fig. 1) and putative recombination indicated by linkage (Supplementary Fig. S7) was not apparent. Genetic cohesion within species may also stem from other causes, such as periodic selection [59] or the *K*/θ ≥ 4 rule derived from population genetic theory [60]. Although a genetic discontinuity below 95% ANI seems to be a rather universal feature in bacteria, cohesion in different species may stem from different microevolutionary processes, and homologous recombination may not be obligatory for species clustering.

### Divergence with spatial distance

**Figure 3:**
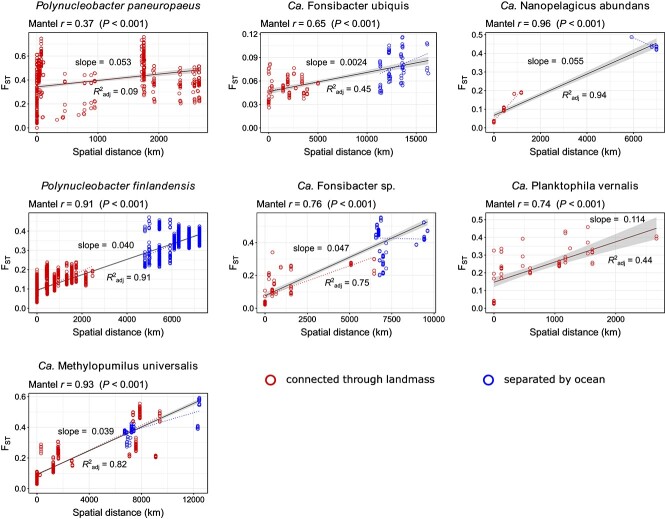
Population differentiation (*F*_ST_) versus spatial distance between habitats for the seven investigated species. Red dots refer to comparisons within Eurasia or North America, blue dots refer to comparisons between Eurasia and North America. The latter are not markedly shifted towards higher F_ST_ values, which suggests that oceans did not pose strong dispersal barriers. Spearman rank correlations across the whole distances assessed through Mantel tests are given on the upper left of each plot. Linear regressions across the whole distances are shown as black lines and the respective *R*^2^_adj_ values are given below each line. These regressions were not used to infer correlations between *F*_ST_ and spatial distance but such correlations are quantified by the Mantel test results. The linear correlations were merely used to display the average increase of F_ST_ with spatial distance within each dataset and quantify the respective slopes (Δ*F*_ST_/1000 km) as given above each line. Linear regressions for only red and only blue datapoints are shown in the respective color as dotted lines.

**Figure 4:**
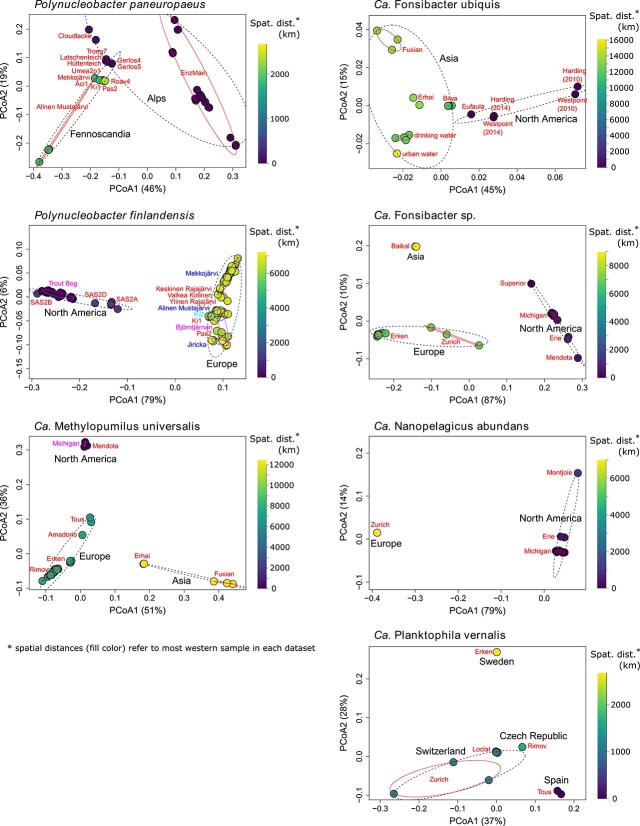
Principal coordinates analyses of *F*_ST_ values. Each dot represents one metagenome. Spatial distances to the most western metagenome for each species are visualized by the color gradient. Metagenomes from the same continent, region, or country are edged by black dotted ellipses. Metagenomes from the same habitat are edged by red ellipses. Habitat names are given in colored font. The percentage of variation explained along each axis is given in the axis titles.

To assess the impact of geographic separation on population differentiation, we analyzed fixation indices (*F*_ST_) from pairwise comparisons of populations versus spatial distance between the respective sampling sites (Fig. 3). F_ST_ is a measure of population divergence that relates inter- to intra-population diversity. For instance, an *F*_ST_ of 0 would mean that the diversity when comparing sequences from different populations would be the same as the diversity within sequences of the same population. The maximum *F*_ST_ of 1 would mean that all polymorphic loci have a fixed allele in one population and an alternative allele in the other population. An *F*_ST_ >0.15 is often considered substantial differentiation [61]. All species showed a significant increase in *F*_ST_ with spatial distance, according to Mantel tests using Spearman rank correlations. The increase appeared rather linear with distance for most of them. This may at first seem surprising if one considers that dispersal when modeled as a diffusion-like process decreases with the square of the distance [13]. Possibly, long-range dispersal might be governed by incremental dispersal events between nearby sites rather than direct dispersal between distant sites (stepping-stone dispersal), which would explain the more linear relationship observed. Yet, the slope tends to be higher at shorter distances (Supplementary Fig. S8), which may reflect a more quadratic decay where direct dispersal between sites is more relevant. We have previously observed a similar pattern when analyzing the genome similarities of *P. pan.* isolates [17]. The observation from that earlier study, that divergence of *P. pan.* does not increase with spatial distance when only longer distances (more than a few hundred km) are considered, was also corroborated in the present work. When comparing the rate of *F*_ST_ increase with spatial distance, it is conspicuous that six of the species were in a similar range (0.039–0.114 Δ*F*_ST_/1000 km), whereas F. ubi. showed a substantially lower divergence (0.0024 Δ*F*_ST_/1000 km). The type strain of this species was isolated from the brackish coastal lagoon of Lake Borgne (salinity of 0.24%) and could be cultivated at salinities up to 0.47% [26]. It is conceivable that higher stress tolerance or a broader niche range of F. ubi. allows the species to better survive dispersal and accordingly explains its low geographic divergence. We do not currently know if it is able to survive a stopover in the oceans to disperse more effectively between continents, but even for the other species, the North Atlantic and North Pacific Oceans did not seem to pose strong dispersal barriers, as no offset to higher *F*_ST_ values was observed for trans-oceanic relative to intra-continental comparisons (Fig. 3). Between Asia and North America, the Bering Strait might facilitate dispersal. Yet, the population structures of M. uni. and F. sp., with metagenomes analyzed from Asia, North America, and Europe, did not show any disproportionally high population divergence between North America and Europe, which are not well connected through inland waters (Fig. 4). Many factors potentially influence microbial dispersal across oceans. It has been shown recently that terrestrial and dust-associated bacteria were more prevalent in the atmospheric community over the Atlantic compared to over the Pacific Ocean [62], which might hint at facilitated dispersal of continental bacteria across the Atlantic. Dust particles are known vectors for bacterial dispersal, and desert dust clouds can be transported long distances, for instance, from Africa to North America [2]. Freshwater bacteria may also hitchhike with migratory waterfowl that cross oceans [63–65]. Although it remains unclear which modes of trans-oceanic dispersal are most relevant, our results suggest that the North Atlantic and North Pacific Oceans are not particularly strong dispersal barriers for the abundant freshwater bacteria analyzed in this study.

### Divergence within habitats

To put the observed population divergence with geographic distance into perspective, we also analyzed spatial and temporal divergence within habitats. We assessed population differentiation along the water column by analyzing metagenomes sampled on the same day but from different depths (Supplementary Fig. S9). Most comparisons (92%) did not show pronounced differentiation (*F*_ST_ < 0.1). Strongest divergence (*F*_ST_ = 0.20) was found in P. ver. between the epilimnion (5 m) and hypolimnion (40–80 m) of Lake Zurich in November 2015. *P. fin.* showed differentiation with F_ST_ values up to 0.16 between samples from oxic surface water (0.25 m) in Lake Björntjärnen as compared to anoxic bottom water (7 m) from the same lake, both collected in September 2018. Overall, divergence across the water column appeared to be minor relative to geographic divergence.

**Figure 5:**
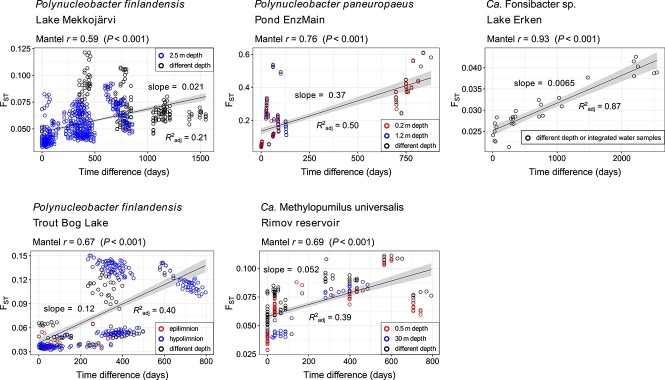
Population differentiation (*F*_ST_) versus time difference between sampling dates for five time series datasets. Colored dots refer to comparisons between samples taken from the same depth. These are not markedly shifted towards lower *F*_ST_ values, which suggests that population differentiation across the water column is minor overall. Spearman rank correlations assessed through Mantel tests are given on the upper left of each plot. Linear regressions are shown as black lines and the respective *R*^2^_adj_ values are given below each line. These regressions were not used to infer correlations between *F*_ST_ and time difference but such correlations are quantified by the Mantel test results. The linear correlations were merely used to display the average increase of *F*_ST_ with time difference within each dataset and quantify the respective slopes (Δ*F*_ST_/1000 days) as given above each line.

For four species in five different habitats, there were extended metagenomic time series (up to 7 years) available [20, 32, 66–69], enabling us to study population differentiation over time. As we had observed for spatial distance, *F*_ST_ increased significantly with time (Fig. 5). Yet, the variability in the rate of this increase was much higher than for geographic divergence. The rate ranged from 0.0065 to 0.37 Δ*F*_ST_/1000 days for the different time series, as compared to a much narrower range of 0.039–0.053 Δ*F*_ST_/1000 km spatial distances for the same four species. The large variability in temporal differentiation points to fundamentally different intra-population dynamics between different species, or possibly also between different populations within the same species. Changes in relative abundance and intra-population diversity with time are shown in Supplementary Fig. S10. The two extremes in terms of temporal dynamics were observed between F. sp. in Lake Erken, which showed very slow and steady divergence with a maximum F_ST_ of 0.043 over 7 years, and *P. pan.* in the Alpine pond EnzMain, with an *F*_ST_ up to 0.54 between two samples retrieved only two months apart. For comparison, maximum *F*_ST_ values between different habitats were 0.55 and 0.76 for F. sp. and *P. pan.*, respectively. Allele frequency distribution changes over time, which are discussed in the next paragraph, provided some hints toward potential explanations for these contrasting rates of differentiation.

### Intra-population dynamics

**Figure 6:**
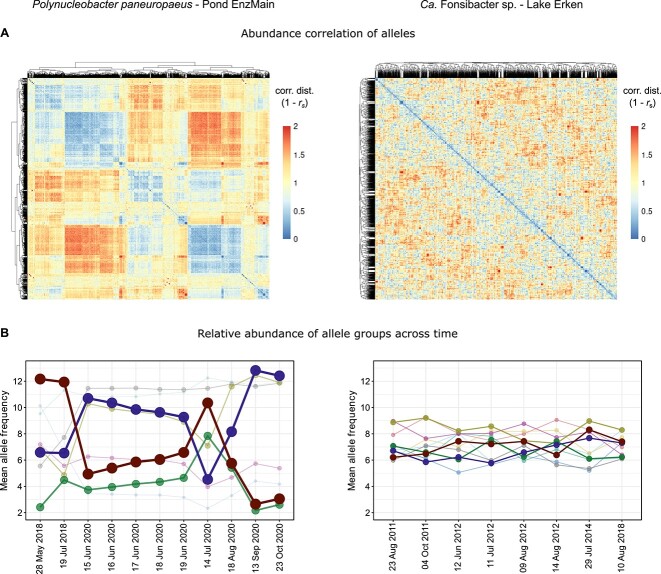
Allele frequency dynamics for two example species with contrasting patterns. (A) Clustering of alleles based on spearman rank correlation coefficients of allele frequencies across time. (B) Alleles were clustered into 9 (*P. pan.*) and 11 (F. sp.) groups representing putative strains based on the correlations shown in A (see also Supplementary Fig. S12). The plots show the abundance of each group of alleles (mean allele frequency) across time. Dot size, line size, and color density are proportional to the number of alleles in the group. Time points are not equidistant and the Lake Erken series spans a significantly longer time period than the pond EnzMain series.

To unravel the contrasting patterns of population differentiation with time between F. sp. and *P. pan.*, we analyzed allele frequency changes in the two time series. The F. sp. allele frequency distribution in Lake Erken hardly changed with time, whereas it was highly dynamic for *P. pan.* in Pond EnzMain (Supplementary Fig. S11). Such contrasting allele frequency dynamics were observed for different populations within a single lake before [20]. To see which alleles show synchronous changes over time and might thus be associated with the same strain, we calculated a correlation matrix for all alleles based on their allele frequencies in the different metagenomes of the time series (Fig. 6A). Alleles seemed to cluster into different groups in *P. pan.*, whereas no clear clustering was apparent in F. sp. After hierarchical cluster analysis, *P. pan.* alleles were grouped into nine and F. sp. into eleven clusters with coherent dynamics (Supplementary Fig. S12), representing subpopulations or putative strains (in the following designated as strains for convenience). The numbers of inferred strains depend on the clustering criteria used and are thus somewhat arbitrary, and the distinction between strains was weak in F. sp. as mentioned above. By tracing the relative abundances of the strains over time, we could nevertheless illustrate the distinct intra-population dynamics between *P. pan*. and F. sp. (Fig. 6B). In *P. pan.*, two strains appeared to alternately dominate the population in the pond. Dominant alleles were swept from the population only to reappear and become dominant again at later times. For example, 117 out of 3977 alleles that were absent (allele frequency = 0/15) in May 2018, became the major allele (frequency ≥ 8/15) in June 2020, were then absent in July 2020, and became the major allele again in September 2020. Sweeps were thus incomplete and transitory in *P. pan.*, meaning that most alleles probably persisted in the population, albeit at times below the detection limit. It seems less likely that alleles were completely lost and reestablished repeatedly through either a novel mutation or recolonization. These observations are in line with the constant-diversity model [70], where a complete sweep of diversity by an over-dominant strain is prevented by strain-specific phage predation constraining and counteracting “blooms” of individual strains (i.e. “kill the winner” [71]). It remains to be tested whether phage predation was responsible for the observed dynamics in *P. pan.*, and it cannot be excluded that complete selective sweeps leading to periodic selection [72] are happening over longer timescales. A selective sweep was suggested for a *Chlorobium* population in Trout Bog Lake, where the initially detected diversity was almost completely purged in 2009 and not reestablished until 2013 [20]. It would be interesting to study the population over even longer timescales to assess the persistence of this sweep. In *P. pan.*, high homologous recombination rates are supposed to unlink different genes in the genome, and putative sweeps might thus be gene-specific rather than genome-wide [20, 73]. Sweeps of diversity and the homogenizing effect of homologous recombination could both constrain diversification and potentially explain the overall low nucleotide diversity in *P. pan*. In contrast to *P. pan.*, the F. sp. population showed an exceptionally slow and continuous evolution with time (Fig. 5), and there were no distinguishable strains with distinct dynamics (Fig. 6, right). The F. sp. population could be viewed either as a consortium of a large number of strains that stably coexist or equally well as one diverse but coherent population. A decisive factor for causing differences in intra-population dynamics may be population size and abundance dynamics. The small changes in composition of the F. sp. population were accompanied by similarly small changes in relative abundance over the seven-year time series, whereas relative abundance varied drastically in *P. pan.* (Supplementary Fig. S10, see Supplementary Text S1 for more details).

Overall, we observed contrasting intra-population dynamics. On the one hand, there are recurring clonal expansions, with different genotypes alternately dominating the population. On the other hand, slow continuous microevolution, where a high diversity of genotypes persists at constant relative abundances. The type of dynamics followed by the populations of a species may have crucial implications for its diversification and the emergence of new species.

### Potential implications for speciation

**Figure 7:**
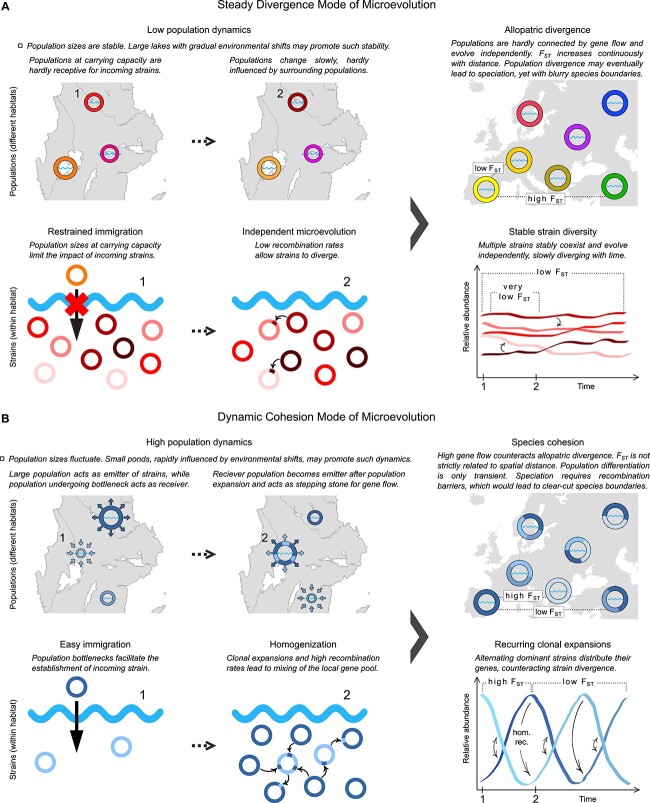
Conceptual models based on two putative modes of microevolution observed in this study.

The contrasting intra-population dynamics and the observed biogeographic patterns suggest that mechanisms of speciation differ between taxa. In F. sp., low differentiation over time in Lake Erken (max. *F*_ST_ of 0.043 over 7 years, Fig. 5) was opposed by comparatively high geographic divergence (max. *F*_ST_ of 0.55 over 9600 km, Fig. 3). Hence, diversification within the species seemed to be dominated by allopatric divergence. Diverse and stable populations within lakes may allow for little gene flow from immigrating, conspecific bacteria. Hence, populations from different lakes may evolve independently of each other and diverge continuously. We term this mechanism of diversification exemplified by F. sp. “Steady Divergence Mode of Microevolution” (Fig. 7A). Speciation events may not be clear-cut, and diversity between species would be rather continuous. The existence of sister species in several metagenomes, indicated by peaks in blast histograms between 90% and 95% identity (Supplementary Figs S1 and S2), corroborates this conclusion. Marine *Ca. Fonsibacter* relatives affiliated with the SAR11 clade showed an even more continuous diversity structure on a global scale [54, 74], possibly owing to the more contiguous nature of marine habitats compared to a generally lower connectivity between inland waters.

*Polynucleobacter paneuropaeus* represented a contrasting example of bacterial microevolution. Population differentiation was high over both time (max. *F*_ST_ of 0.61 over 2.5 years, Fig. 5) and space (max. *F*_ST_ of 0.76 over 2700 km, Fig. 3), but also highly fluctuating with time and spatial distances. Low strain diversity within habitats, high homologous recombination rates, and recurring clonal expansions may allow immigrating, conspecific bacteria to substantially impact resident populations. This results in extensive gene flow that counteracts geographic divergence, as has been suggested earlier [17]. We summarize these mechanisms under the “Dynamic Cohesion Mode of Microevolution” (Fig. 7B). For speciation to occur under these circumstances, a lineage might be required to transition to a new ecological niche, for instance, a different habitat type, which would result in spatial separation and impaired recombination with the ancestral lineage. Such a process of bacterial speciation, which could be triggered by the acquisition of novel functions through horizontal gene transfer, has been hypothesized previously [75]. These mechanisms would lead to clear-cut genetic boundaries between ecologically distinct species. The pronounced genetic discontinuity between *Polynucleobacter* species ([31] and this study) accompanied by ecological distinguishability [30], corroborates such a model of speciation for *Polynucleobacter*.

Acknowledging differing models of speciation as described above may help to better understand bacterial evolution and the ecological meaning of bacterial species.

## Conclusion

All investigated taxa formed sequence-discrete species across the studied geographic scales, suggesting that global freshwater bacterial diversity is clustered into genetically coherent units. Mapped reads peaking consistently at >95% identity corroborated the usefulness of the widely used 95% ANI threshold for species delineation. Still, divergence within species differed considerably, pointing to distinct microevolutionary mechanisms that shape the diversity within different taxa. Population differentiation increased with spatial distance in all species, although major dispersal barriers were not apparent, and oceans did not seem to considerably limit dispersal. Species with broad habitat ranges may be dispersed particularly well, as suggested by the minimal geographic divergence of the salt-tolerant *Ca.* Fonsibacter ubiquis. Population structuring along water column depth gradients was mostly minor. In contrast, we observed striking differences between populations in their temporal dynamics. The divergence of a *Ca.* Fonsibacter sp. population in Lake Erken was considerably lower over 7 years than that seen between any two populations from different lakes, whereas *Polynucleobacter paneuropaeus* from an Alpine pond reached a similar divergence as seen over pan-European scales within only 2 months. We thus propose two contrasting models of microevolution. (i) Steady Divergence: As suggested in *Fonsibacter* sp., high intra-population diversity leads to stable progression of populations, which evolve more independently from conspecific populations of other habitats. Geographic separation and continuous temporal divergence might be sufficient for speciation to progress, yet, result in more blurry species boundaries. (ii) Dynamic Cohesion: As observed in *P. paneuropaeus*, recurring clonal expansions result in low-diversity populations at any given time point. Low intra-population diversity combined with high recombination rates allow for effective gene flow between populations. Speciation might require ecological differentiation and gives rise to clear-cut species boundaries.

## Supplementary Material

SupplFigS1_blast_histograms_ANIr95_wrae113

SupplFigS2_excluded_metagenomes_blast_histograms_wrae113

SupplFigS3_ANIr95_with_and_without_home_wrae113

SupplFigS4_test_Hnoncorr_wrae113

SupplFigS5_maps_all_species_and_separate_wrae113

SupplFigS6_mapped_bases_wrae113

SupplFigS7_linkage_revised_wrae113

SupplFigS8_FSTvsSpatDist_500km_wrae113

SupplFigS9_FST_between_depths_wrae113

SupplFigS10_time_series_dynamics_revised_wrae113

SupplFigS11_allele_freq_histograms_wrae113

SupplFigS12_hclust_group_alleles_wrae113

SupplTableS1_initial_ref_genomes_wrae113

SupplTableS2_newly_generated_metagenomes_wrae113

SupplTableS3_metagenomes_overview_wrae113

SupplTableS4_population_genomics

SupplTableS5_strain_diversity_test_dataset

SupplTextS1_time_series_dynamics_wrae113

## Data Availability

Metagenomic data obtained for this study is available in the NCBI database at https://www.ncbi.nlm.nih.gov under BioProject accession PRJNA965924, SRA accessions SRR24390586-SRR24390649. Custom scripts used in this study are available on GitHub at https://github.com/thr44pw00d/population-structure.
