# Supplementary material for: Geographic population structure and distinct intra-population dynamics of globally abundant freshwater bacteria
Source: ISME J. 2024 Jul 3;18(1):wrae113. doi: 10.1093/ismejo/wrae113 (PMC11283720; doi:10.1093/ismejo/wrae113)
Supplement: SupplFigS2_excluded_metagenomes_blast_histograms_wrae113 [file supplfigs2_excluded_metagenomes_blast_histograms_wrae113.pdf]

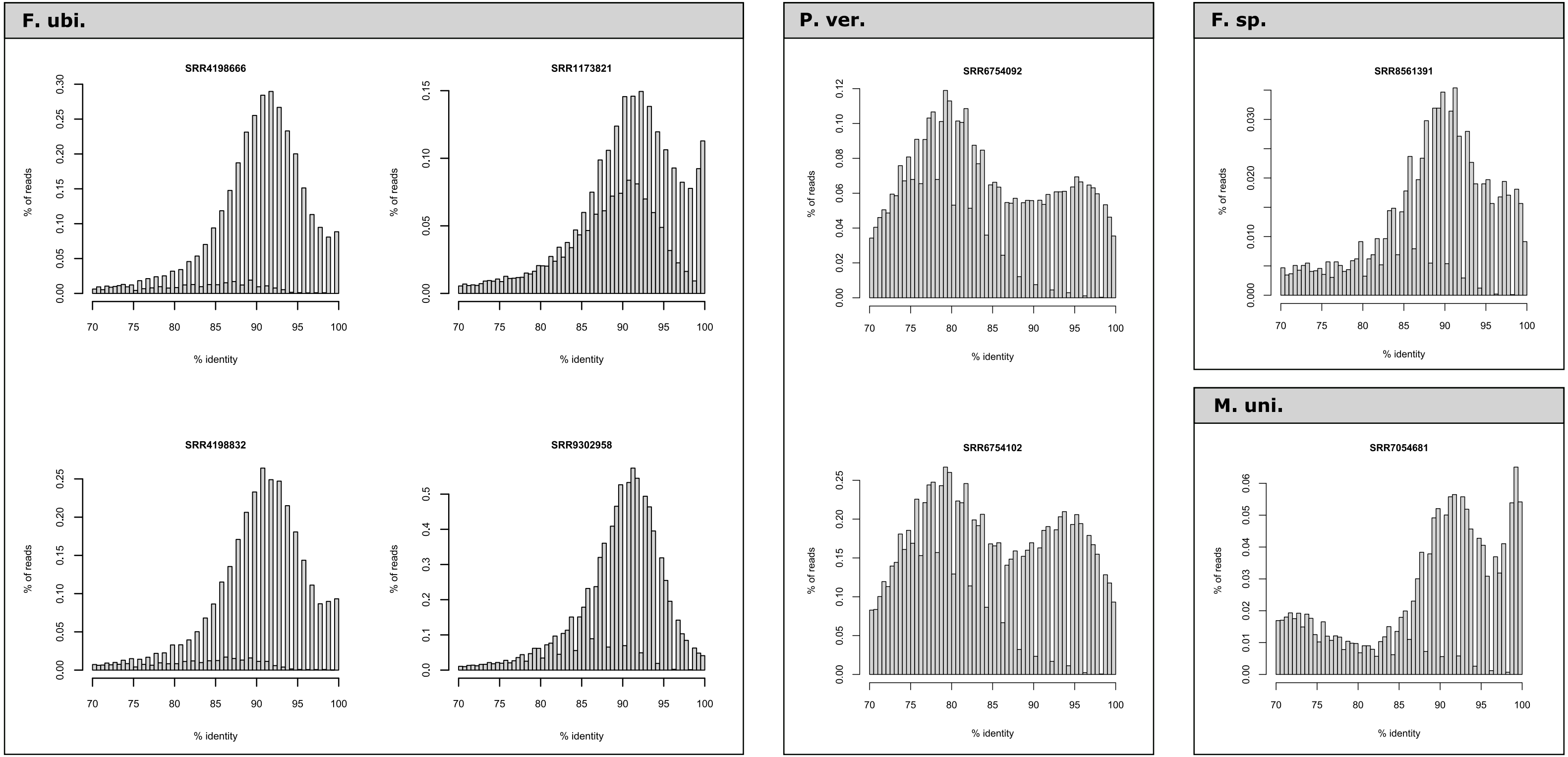

Suppl. Fig. S2: Omitted metagenomes due to presence of sister species. These eight metagenomes were excluded from analyses of the respective species as the number of reads mapped with 90-95% identity was higher than the number of reads with >95% identity, i.e. a substantial fraction of the latter reads may have originated from a sister species rather than the species of interest.
