# Supplementary material for: Geographic population structure and distinct intra-population dynamics of globally abundant freshwater bacteria
Source: ISME J. 2024 Jul 3;18(1):wrae113. doi: 10.1093/ismejo/wrae113 (PMC11283720; doi:10.1093/ismejo/wrae113)
Supplement: SupplFigS3_ANIr95_with_and_without_home_wrae113 [file supplfigs3_anir95_with_and_without_home_wrae113.pdf]

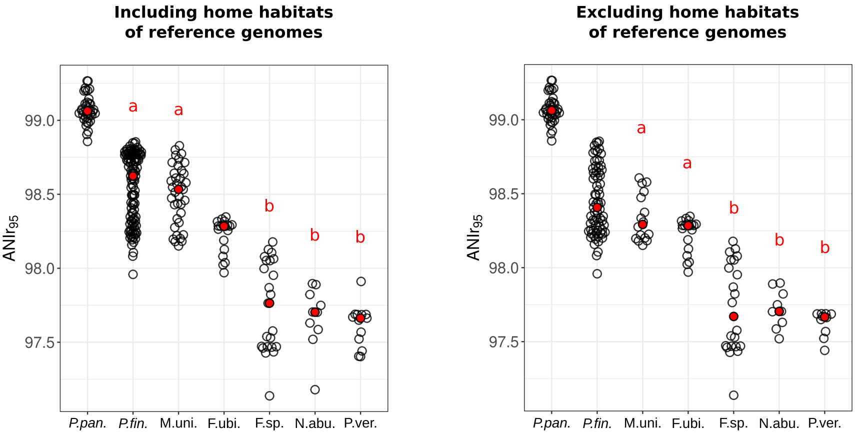

Suppl. Fig. S3: $ANIr_{95}$ values for the seven species when metagenomes sampled from the habitat where the reference genome was obtained from are included (left) and excluded (right). Each dot refers to one metagenome. Red dots show the medians. Within each plot, pairs that are not significantly different ($p > 0.05$) according to Wilcoxon-Mann-Whitmey Rank Sum test are marked with a common letter.
