# Supplementary material for: Geographic population structure and distinct intra-population dynamics of globally abundant freshwater bacteria
Source: ISME J. 2024 Jul 3;18(1):wrae113. doi: 10.1093/ismejo/wrae113 (PMC11283720; doi:10.1093/ismejo/wrae113)
Supplement: SupplFigS4_test_Hnoncorr_wrae113 [file supplfigs4_test_hnoncorr_wrae113.pdf]

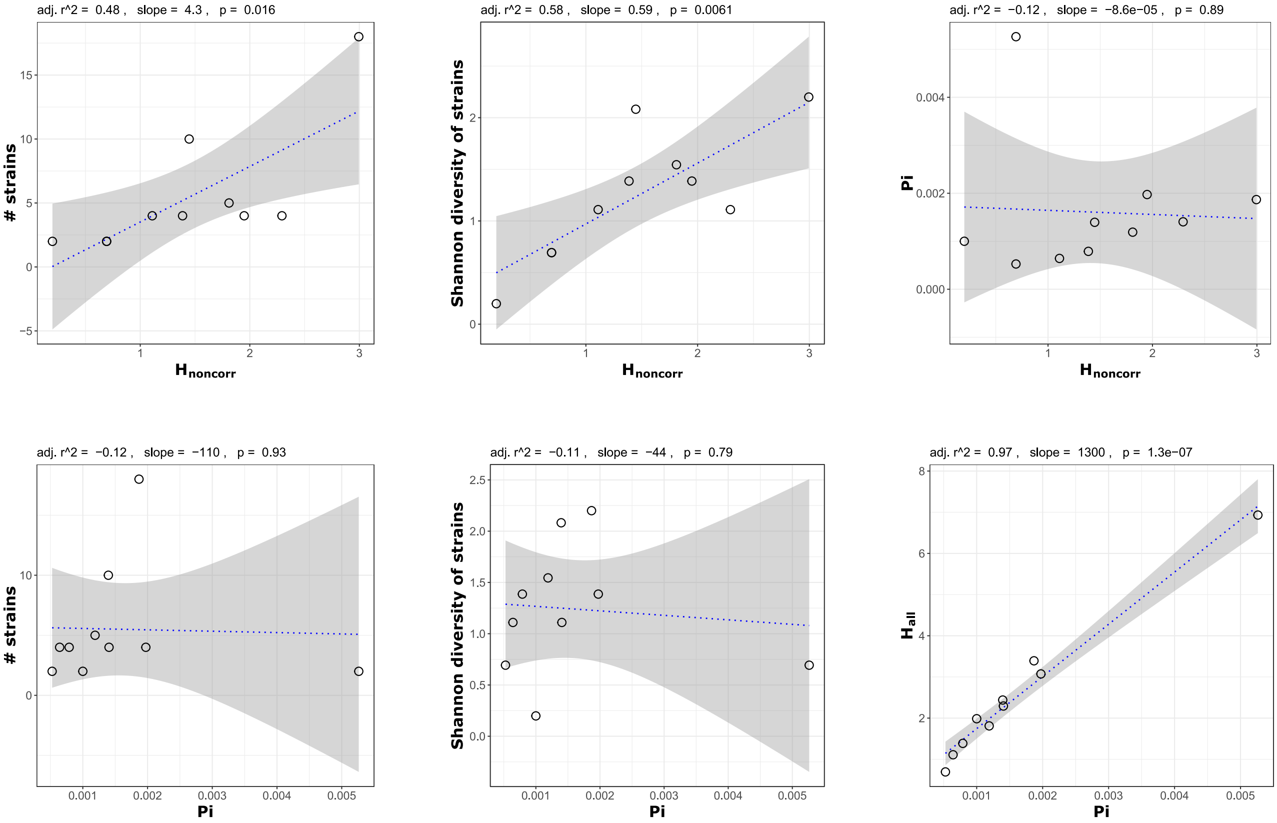

Suppl. Fig. S4: Comparison of different diversity measures in test dataset. The Shannon entropy of polymorphic loci with non-correlated allele frequencies ($H_{noncorr}$) correlates better with the number of strains than Pi. The latter diversity measure is strongly correlated with the Shannon entropy of all polymorphic loci (H_{all}), i.e. it increases with the number of polymorphic loci regardless of correlation among them.
