# Supplementary material for: Geographic population structure and distinct intra-population dynamics of globally abundant freshwater bacteria
Source: ISME J. 2024 Jul 3;18(1):wrae113. doi: 10.1093/ismejo/wrae113 (PMC11283720; doi:10.1093/ismejo/wrae113)
Supplement: SupplFigS5_maps_all_species_and_separate_wrae113 [file supplfigs5_maps_all_species_and_separate_wrae113.pdf]

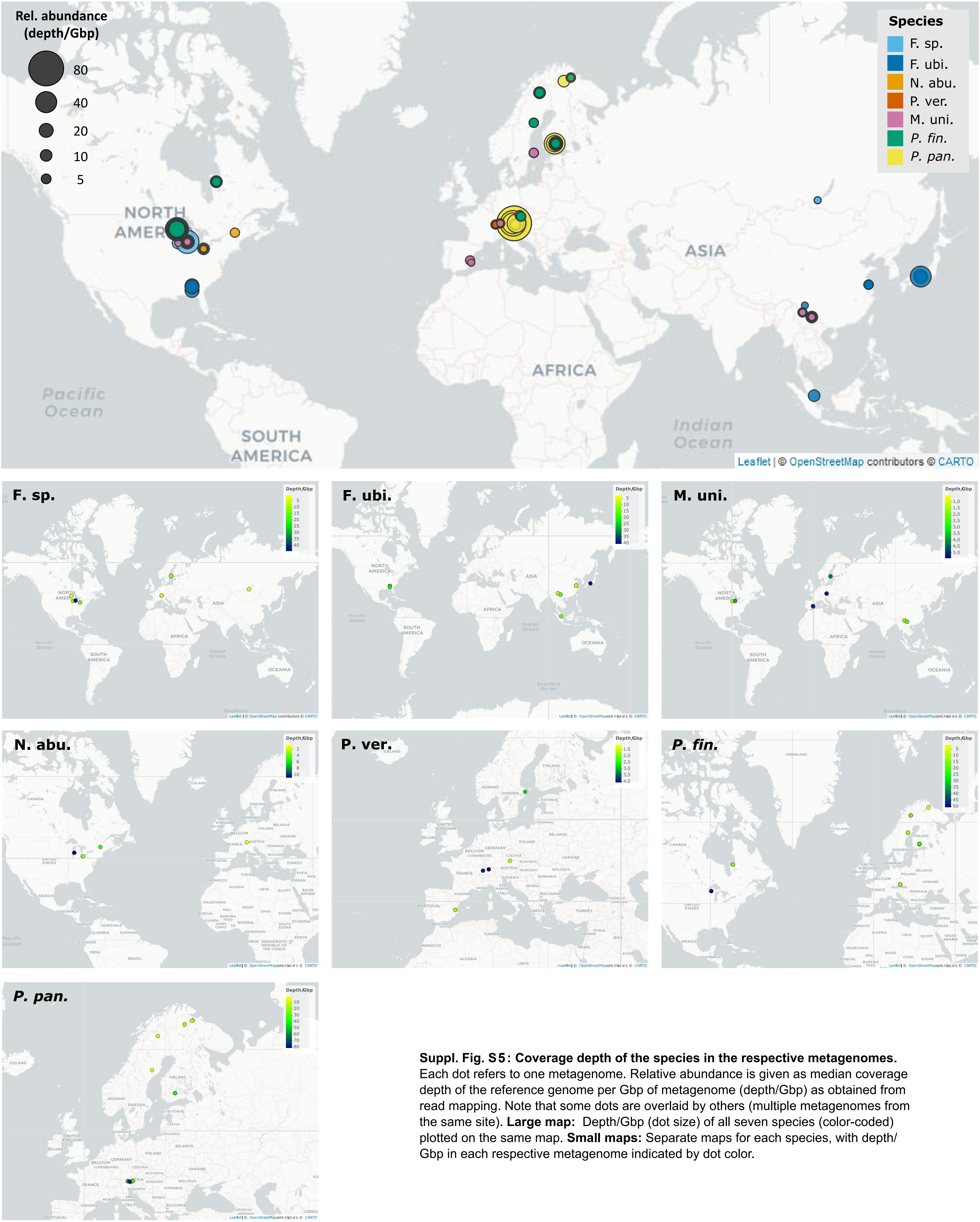

Suppl. Fig. S5: Coverage depth of the species in the respective metagenomes. Each dot refers to one metagenome. Relative abundance is given as median coverage depth of the reference genome per Gbp of metagenome (depth/Gbp) as obtained from read mapping. Note that some dots are overlaid by others (multiple metagenomes from the same site). **Large map:** Depth/Gbp (dot size) of all seven species (color-coded) plotted on the same map. **Small maps:** Separate maps for each species, with depth/Gbp in each respective metagenome indicated by dot color.
