# Supplementary material for: Geographic population structure and distinct intra-population dynamics of globally abundant freshwater bacteria
Source: ISME J. 2024 Jul 3;18(1):wrae113. doi: 10.1093/ismejo/wrae113 (PMC11283720; doi:10.1093/ismejo/wrae113)
Supplement: SupplFigS6_mapped_bases_wrae113 [file supplfigs6_mapped_bases_wrae113.pdf]

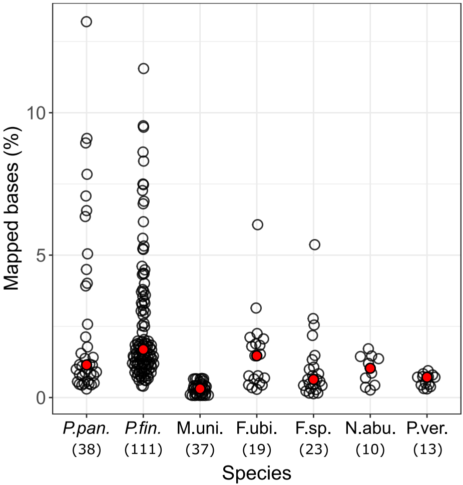

Suppl. Fig. S6: Percentage of metagenome bases mapped to the reference genomes. Each dot refers to one metagenome. Median values are shown in red. The number of metagenomes for each species is given in brackets under the x-axis.
