# Supplementary material for: Geographic population structure and distinct intra-population dynamics of globally abundant freshwater bacteria
Source: ISME J. 2024 Jul 3;18(1):wrae113. doi: 10.1093/ismejo/wrae113 (PMC11283720; doi:10.1093/ismejo/wrae113)
Supplement: SupplFigS7_linkage_revised_wrae113 [file supplfigs7_linkage_revised_wrae113.pdf]

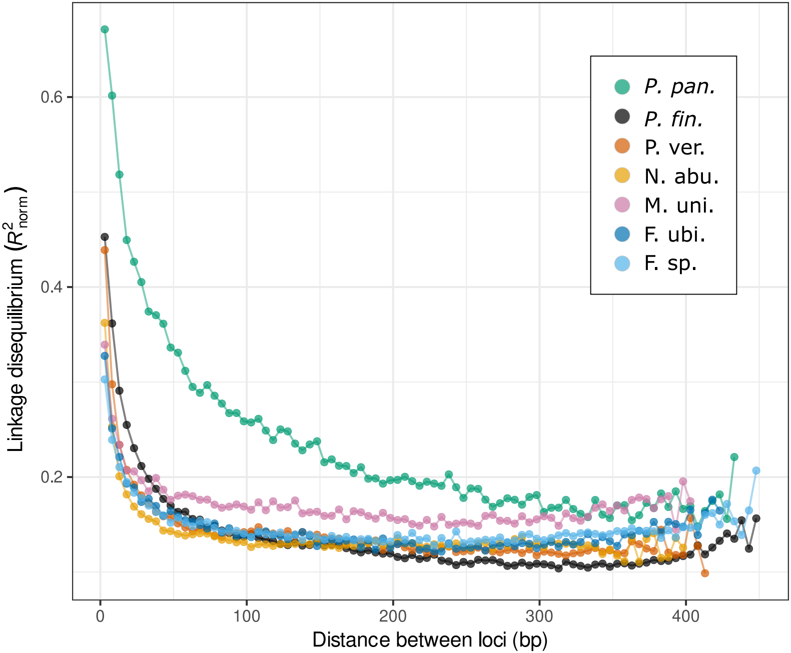

Suppl. Fig. S7.: Linkage between polymorphic loci. The normalized correlation coefficient between pairs of loci (r2norm) was averaged over different pairs with similar distance between the loci, using a 5 bp window. Only windows with at least 20 values were plotted. Higher r2norm values suggest that loci at the given distance were more likely inherited together.
