# Supplementary material for: Geographic population structure and distinct intra-population dynamics of globally abundant freshwater bacteria
Source: ISME J. 2024 Jul 3;18(1):wrae113. doi: 10.1093/ismejo/wrae113 (PMC11283720; doi:10.1093/ismejo/wrae113)
Supplement: SupplFigS9_FST_between_depths_wrae113 [file supplfigs9_fst_between_depths_wrae113.pdf]

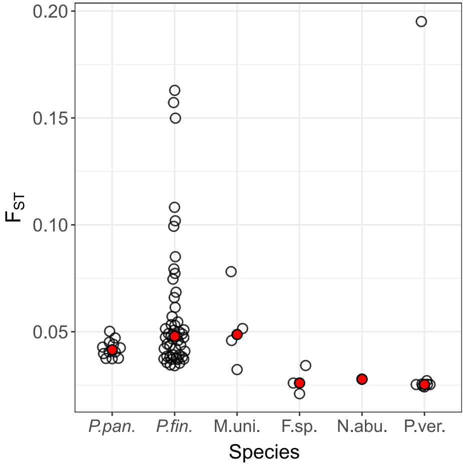

Suppl. Fig. S9.: Population differentiation within the water column. FST between samples taken from the same habitat at the same time but from different depths. Median values are shown in red.
