# Supplementary material for: Geographic population structure and distinct intra-population dynamics of globally abundant freshwater bacteria
Source: ISME J. 2024 Jul 3;18(1):wrae113. doi: 10.1093/ismejo/wrae113 (PMC11283720; doi:10.1093/ismejo/wrae113)
Supplement: SupplFigS10_time_series_dynamics_revised_wrae113 [file supplfigs10_time_series_dynamics_revised_wrae113.pdf]

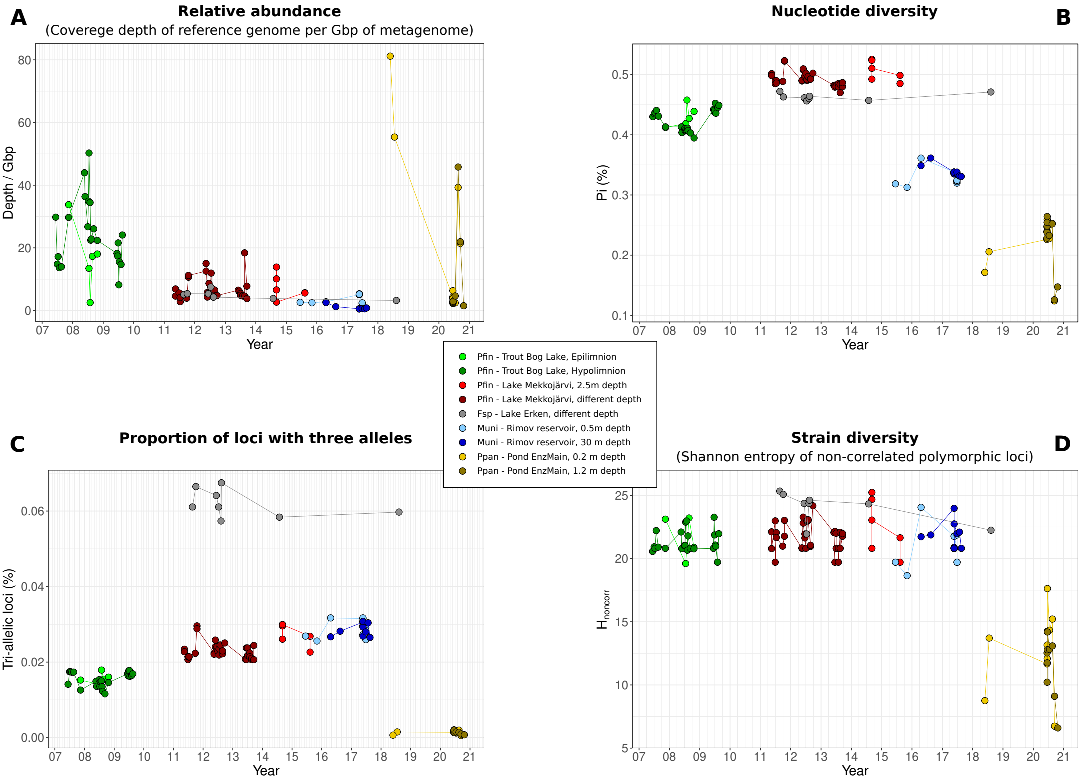

Suppl. Fig. S10.: Population dynamics in time series metagenomes. Relative abundance inferred from read mapping (A) and different diversity measures (B, C, D) in time series for four species in five different habitats. Data points for metagenomes from the same habitat but sampled from different depths are depicted by similar color but different brightness.
