# Supplementary material for: Geographic population structure and distinct intra-population dynamics of globally abundant freshwater bacteria
Source: ISME J. 2024 Jul 3;18(1):wrae113. doi: 10.1093/ismejo/wrae113 (PMC11283720; doi:10.1093/ismejo/wrae113)
Supplement: SupplFigS1_blast_histograms_ANIr95_wrae113 [file supplfigs1_blast_histograms_anir95_wrae113.pdf]

Suppl. Fig. S1: Histograms of all metagenomes per species mapped to the respective reference genome by Blast. The $ANIr_{95}$ value is given and indicated by a blue dashed line for each histogram. The histograms are sorted by increasing spatial distance between the sampling site of the metagenome and the home habitat of the reference genome. The respective distances are given in km next to the metagenome name on top of each histogram.

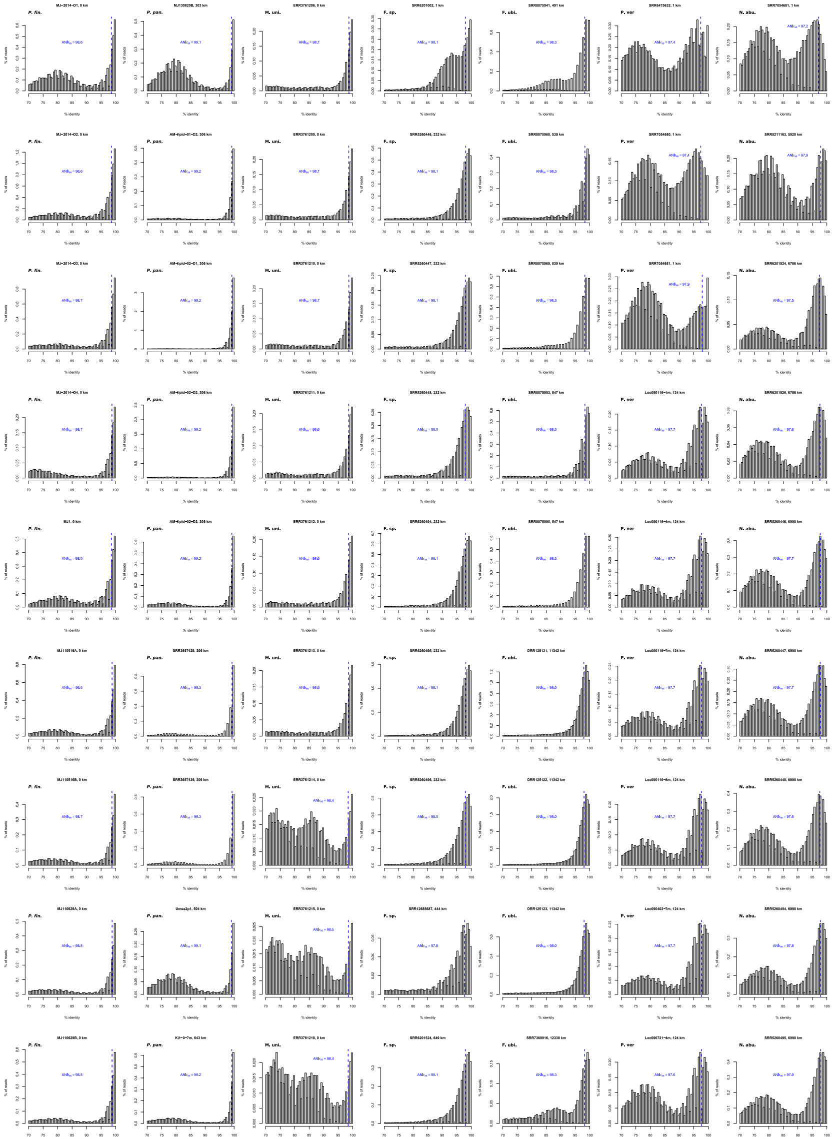

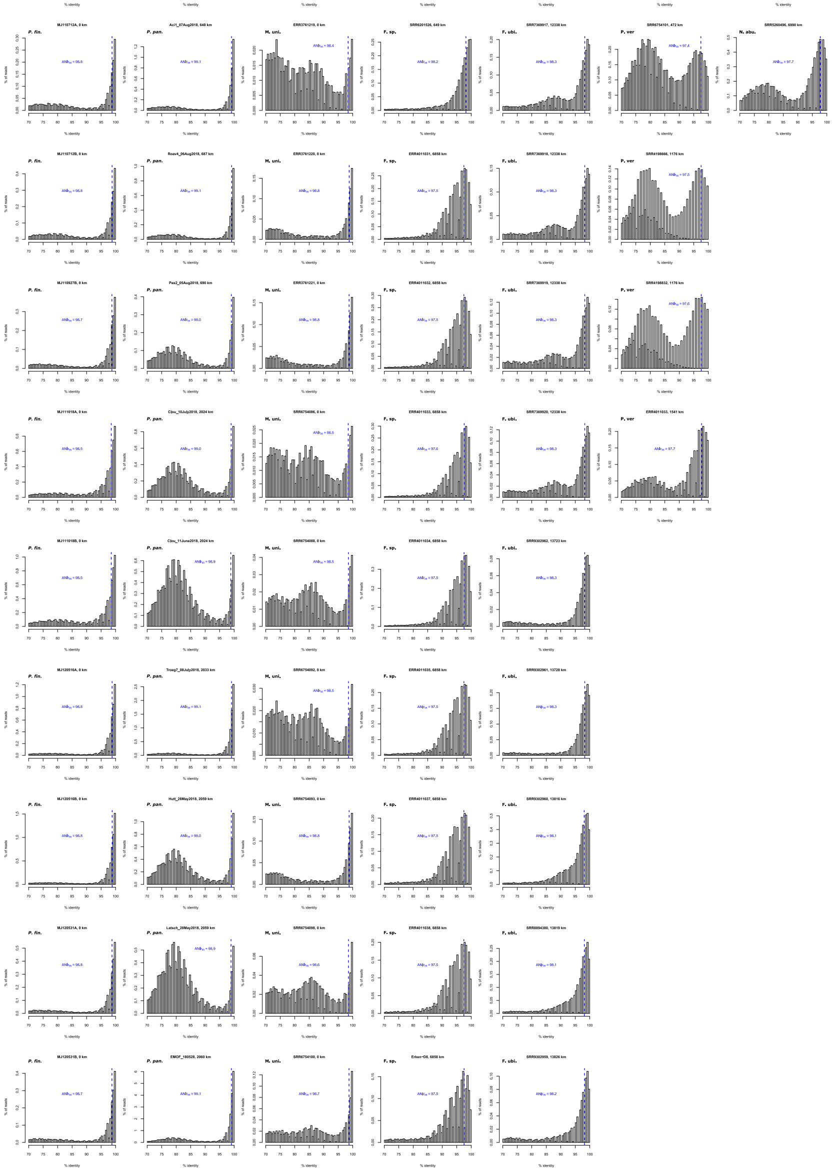

% identity
% of reads
P. fin.
MJ110712A, 0 km
ANIr$_{95}$ = 98.8
MJ110712B, 0 km
ANIr$_{95}$ = 98.8
MJ110927B, 0 km
ANIr$_{95}$ = 98.7
MJ111018A, 0 km
ANIr$_{95}$ = 98.5
MJ111018B, 0 km
ANIr$_{95}$ = 98.5
MJ120516A, 0 km
ANIr$_{95}$ = 98.8
MJ120516B, 0 km
ANIr$_{95}$ = 98.8
MJ120531A, 0 km
ANIr$_{95}$ = 98.8
MJ120531B, 0 km
ANIr$_{95}$ = 98.7
P. pan.
Aci1_07Aug2018, 648 km
ANIr$_{95}$ = 99.1
Roav4_06Aug2018, 687 km
ANIr$_{95}$ = 99.1
Pas2_05Aug2018, 690 km
ANIr$_{95}$ = 99.0
Clou_10July2018, 2024 km
ANIr$_{95}$ = 99.0
Clou_11June2018, 2024 km
ANIr$_{95}$ = 98.9
Troeg7_08July2018, 2033 km
ANIr$_{95}$ = 99.1
Hutt_28May2018, 2059 km
ANIr$_{95}$ = 99.0
Latsch_28May2018, 2059 km
ANIr$_{95}$ = 98.9
EMOF_180528, 2060 km
ANIr$_{95}$ = 99.1
M. uni.
ERR3761219, 0 km
ANIr$_{95}$ = 98.4
ERR3761220, 0 km
ANIr$_{95}$ = 98.8
ERR3761221, 0 km
ANIr$_{95}$ = 98.8
SRR6754086, 0 km
ANIr$_{95}$ = 98.5
SRR6754088, 0 km
ANIr$_{95}$ = 98.5
SRR6754092, 0 km
ANIr$_{95}$ = 98.5
SRR6754093, 0 km
ANIr$_{95}$ = 98.8
SRR6754098, 0 km
ANIr$_{95}$ = 98.6
SRR6754100, 0 km
ANIr$_{95}$ = 98.7
F. sp.
SRR6201526, 649 km
ANIr$_{95}$ = 98.2
ERR4011031, 6858 km
ANIr$_{95}$ = 97.5
ERR4011032, 6858 km
ANIr$_{95}$ = 97.5
ERR4011033, 6858 km
ANIr$_{95}$ = 97.6
ERR4011034, 6858 km
ANIr$_{95}$ = 97.5
ERR4011035, 6858 km
ANIr$_{95}$ = 97.5
ERR4011037, 6858 km
ANIr$_{95}$ = 97.5
ERR4011038, 6858 km
ANIr$_{95}$ = 97.5
Erken-D8, 6858 km
ANIr$_{95}$ = 97.5
F. ubi.
SRR7369917, 12338 km
ANIr$_{95}$ = 98.3
SRR7369918, 12338 km
ANIr$_{95}$ = 98.3
SRR7369919, 12338 km
ANIr$_{95}$ = 98.3
SRR7369920, 12338 km
ANIr$_{95}$ = 98.3
SRR9302962, 13723 km
ANIr$_{95}$ = 98.3
SRR9302961, 13728 km
ANIr$_{95}$ = 98.3
SRR9302960, 13816 km
ANIr$_{95}$ = 98.1
SRR8894380, 13819 km
ANIr$_{95}$ = 98.1
SRR9302959, 13826 km
ANIr$_{95}$ = 98.2
P. ver
SRR6754101, 472 km
ANIr$_{95}$ = 97.4
SRR4198666, 1176 km
ANIr$_{95}$ = 97.5
SRR4198832, 1176 km
ANIr$_{95}$ = 97.6
ERR4011033, 1541 km
ANIr$_{95}$ = 97.7
N. abu.
SRR5260496, 6990 km
ANIr$_{95}$ = 97.7

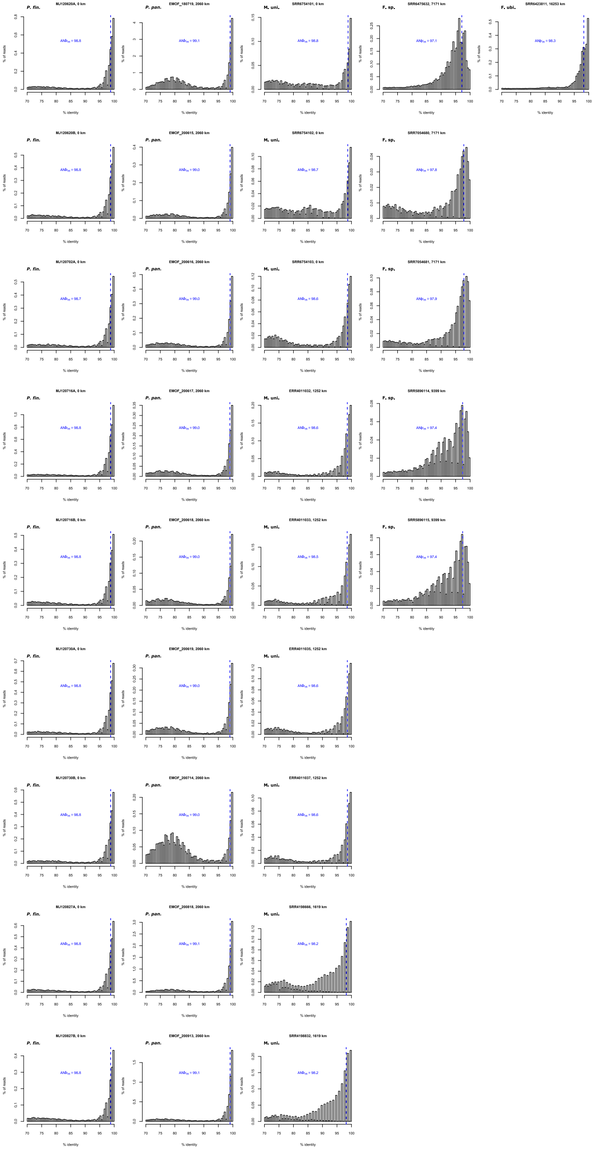
MJ120620A, 0 km
P. fin.
ANIr95 = 98.8
EMOF_180719, 2060 km
P. pan.
ANIr95 = 99.1
SRR6754101, 0 km
M. uni.
ANIr95 = 98.8
SRR6475632, 7171 km
F. sp.
ANIr95 = 97.1
SRR6423811, 16253 km
F. ubi.
ANIr95 = 98.3
MJ120620B, 0 km
P. fin.
ANIr95 = 98.8
EMOF_200615, 2060 km
P. pan.
ANIr95 = 99.0
SRR6754102, 0 km
M. uni.
ANIr95 = 98.7
SRR7054680, 7171 km
F. sp.
ANIr95 = 97.8
MJ120702A, 0 km
P. fin.
ANIr95 = 98.7
EMOF_200616, 2060 km
P. pan.
ANIr95 = 99.0
SRR6754103, 0 km
M. uni.
ANIr95 = 98.6
SRR7054681, 7171 km
F. sp.
ANIr95 = 97.9
MJ120716A, 0 km
P. fin.
ANIr95 = 98.8
EMOF_200617, 2060 km
P. pan.
ANIr95 = 99.0
ERR4011032, 1252 km
M. uni.
ANIr95 = 98.6
SRR5896114, 9399 km
F. sp.
ANIr95 = 97.4
MJ120716B, 0 km
P. fin.
ANIr95 = 98.8
EMOF_200618, 2060 km
P. pan.
ANIr95 = 99.0
ERR4011033, 1252 km
M. uni.
ANIr95 = 98.5
SRR5896115, 9399 km
F. sp.
ANIr95 = 97.4
MJ120730A, 0 km
P. fin.
ANIr95 = 98.8
EMOF_200619, 2060 km
P. pan.
ANIr95 = 99.0
ERR4011035, 1252 km
M. uni.
ANIr95 = 98.6
MJ120730B, 0 km
P. fin.
ANIr95 = 98.8
EMOF_200714, 2060 km
P. pan.
ANIr95 = 99.0
ERR4011037, 1252 km
M. uni.
ANIr95 = 98.6
MJ120827A, 0 km
P. fin.
ANIr95 = 98.8
EMOF_200818, 2060 km
P. pan.
ANIr95 = 99.1
SRR4198666, 1619 km
M. uni.
ANIr95 = 98.2
MJ120827B, 0 km
P. fin.
ANIr95 = 98.8
EMOF_200913, 2060 km
P. pan.
ANIr95 = 99.1
SRR4198832, 1619 km
M. uni.
ANIr95 = 98.2
% of reads
% identity

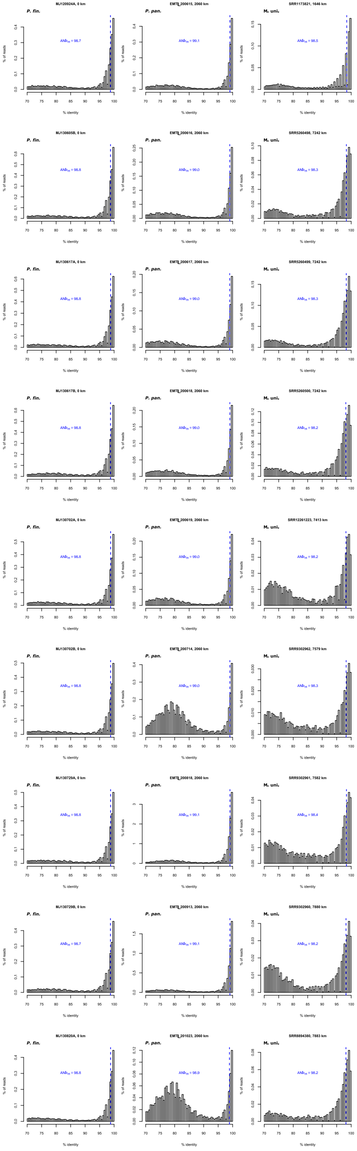
MJ120924A, 0 km
P. fin.
ANIr95 = 98.7
EMTL_200615, 2060 km
P. pan.
ANIr95 = 99.1
SRR1173821, 1646 km
M. uni.
ANIr95 = 98.5
MJ130605B, 0 km
P. fin.
ANIr95 = 98.8
EMTL_200616, 2060 km
P. pan.
ANIr95 = 99.0
SRR5260498, 7242 km
M. uni.
ANIr95 = 98.3
MJ130617A, 0 km
P. fin.
ANIr95 = 98.8
EMTL_200617, 2060 km
P. pan.
ANIr95 = 99.0
SRR5260499, 7242 km
M. uni.
ANIr95 = 98.3
MJ130617B, 0 km
P. fin.
ANIr95 = 98.8
EMTL_200618, 2060 km
P. pan.
ANIr95 = 99.0
SRR5260500, 7242 km
M. uni.
ANIr95 = 98.2
MJ130702A, 0 km
P. fin.
ANIr95 = 98.8
EMTL_200619, 2060 km
P. pan.
ANIr95 = 99.0
SRR12261223, 7413 km
M. uni.
ANIr95 = 98.2
MJ130702B, 0 km
P. fin.
ANIr95 = 98.8
EMTL_200714, 2060 km
P. pan.
ANIr95 = 99.0
SRR9302962, 7579 km
M. uni.
ANIr95 = 98.3
MJ130729A, 0 km
P. fin.
ANIr95 = 98.8
EMTL_200818, 2060 km
P. pan.
ANIr95 = 99.1
SRR9302961, 7582 km
M. uni.
ANIr95 = 98.4
MJ130729B, 0 km
P. fin.
ANIr95 = 98.7
EMTL_200913, 2060 km
P. pan.
ANIr95 = 99.1
SRR9302960, 7880 km
M. uni.
ANIr95 = 98.2
MJ130820A, 0 km
P. fin.
ANIr95 = 98.8
EMTL_201023, 2060 km
P. pan.
ANIr95 = 98.9
SRR8894380, 7883 km
M. uni.
ANIr95 = 98.2
% of reads
% identity

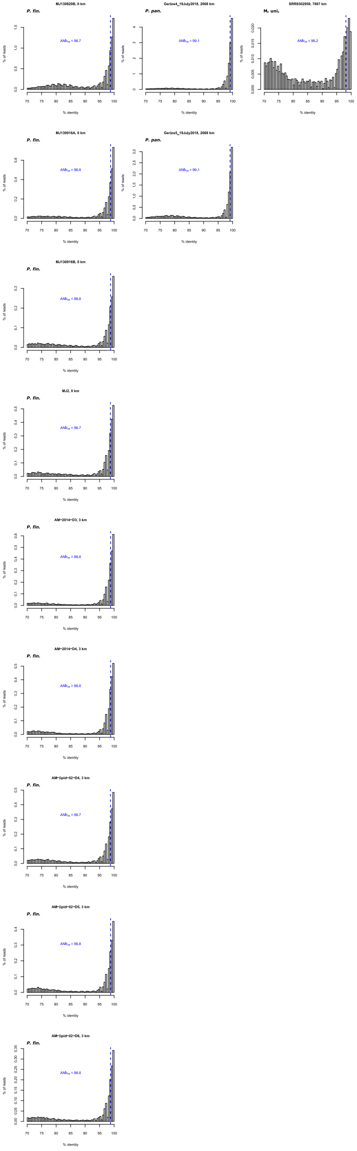
MJ130820B, 0 km
P. fin.
ANIr95 = 98.7
Ger|os4_19July2018, 2068 km
P. pan.
ANIr95 = 99.1
SRR9302959, 7887 km
M. uni.
ANIr95 = 98.2
MJ130916A, 0 km
P. fin.
ANIr95 = 98.8
Ger|os5_19July2018, 2068 km
P. pan.
ANIr95 = 99.1
MJ130916B, 0 km
P. fin.
ANIr95 = 98.8
MJ2, 0 km
P. fin.
ANIr95 = 98.7
AM−2014−D3, 3 km
P. fin.
ANIr95 = 98.8
AM−2014−D4, 3 km
P. fin.
ANIr95 = 98.8
AM−lipid−02−D4, 3 km
P. fin.
ANIr95 = 98.7
AM−lipid−02−D5, 3 km
P. fin.
ANIr95 = 98.8
AM−lipid−02−D6, 3 km
P. fin.
ANIr95 = 98.8
% of reads
% identity

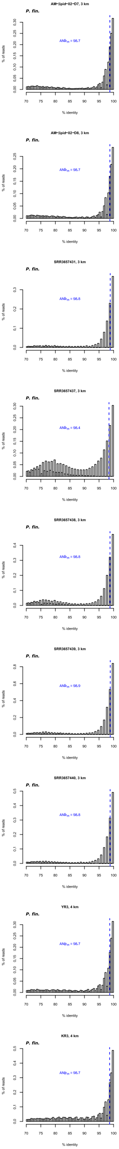
AM−lipid−02−D7, 3 km
P. fin.
ANIr95 = 98.7
AM−lipid−02−D8, 3 km
P. fin.
ANIr95 = 98.7
SRR3657431, 3 km
P. fin.
ANIr95 = 98.8
SRR3657437, 3 km
P. fin.
ANIr95 = 98.4
SRR3657438, 3 km
P. fin.
ANIr95 = 98.8
SRR3657439, 3 km
P. fin.
ANIr95 = 98.9
SRR3657440, 3 km
P. fin.
ANIr95 = 98.8
YR3, 4 km
P. fin.
ANIr95 = 98.7
KR3, 4 km
P. fin.
ANIr95 = 98.7
% of reads
% identity

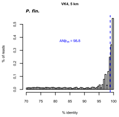
VK4, 5 km
P. fin.
$ANIr_{95}$ = 98.8
% of reads
0.0 0.1 0.2 0.3 0.4 0.5
70 75 80 85 90 95 100
% identity

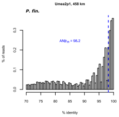
Umea2p1, 458 km
P. fin.
$ANIr_{95}$ = 98.2
% of reads
0.0 0.1 0.2 0.3
70 75 80 85 90 95 100
% identity

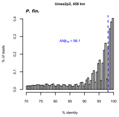
Umea2p2, 458 km
P. fin.
$ANIr_{95}$ = 98.1
% of reads
0.0 0.1 0.2 0.3 0.4
70 75 80 85 90 95 100
% identity

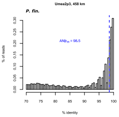
Umea2p3, 458 km
P. fin.
$ANIr_{95}$ = 98.5
% of reads
0.00 0.05 0.10 0.15 0.20 0.25 0.30
70 75 80 85 90 95 100
% identity

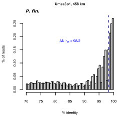
Umea3p1, 458 km
P. fin.
$ANIr_{95}$ = 98.2
% of reads
0.00 0.05 0.10 0.15 0.20 0.25
70 75 80 85 90 95 100
% identity

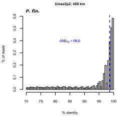
Umea3p2, 458 km
P. fin.
$ANIr_{95}$ = 98.6
% of reads
0.0 0.1 0.2 0.3 0.4 0.5 0.6
70 75 80 85 90 95 100
% identity

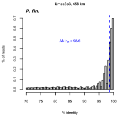
Umea3p3, 458 km
P. fin.
$ANIr_{95}$ = 98.6
% of reads
0.0 0.1 0.2 0.3 0.4 0.5 0.6 0.7
70 75 80 85 90 95 100
% identity

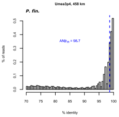
Umea3p4, 458 km
P. fin.
$ANIr_{95}$ = 98.7
% of reads
0.0 0.1 0.2 0.3 0.4 0.5
70 75 80 85 90 95 100
% identity

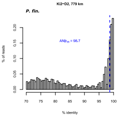
Ki2−D2, 779 km
P. fin.
$ANIr_{95}$ = 98.7
% of reads
0.00 0.05 0.10 0.15 0.20
70 75 80 85 90 95 100
% identity

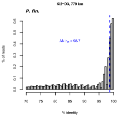
Ki2−D3, 779 km
P. fin.
ANIr95 = 98.7
% of reads
% identity

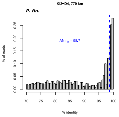
Ki2−D4, 779 km
P. fin.
ANIr95 = 98.7
% of reads
% identity

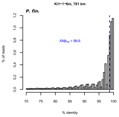
Ki1−1−6m, 781 km
P. fin.
ANIr95 = 98.6
% of reads
% identity

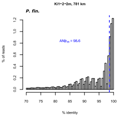
Ki1−2−2m, 781 km
P. fin.
ANIr95 = 98.6
% of reads
% identity

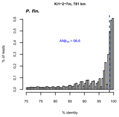
Ki1−2−7m, 781 km
P. fin.
ANIr95 = 98.6
% of reads
% identity

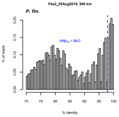
Pas2_05Aug2018, 949 km
P. fin.
ANIr95 = 98.0
% of reads
% identity

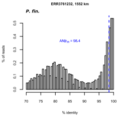
ERR3761232, 1552 km
P. fin.
ANIr95 = 98.4
% of reads
% identity

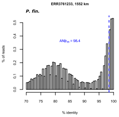
ERR3761233, 1552 km
P. fin.
ANIr95 = 98.4
% of reads
% identity

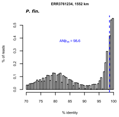
ERR3761234, 1552 km
P. fin.
ANIr95 = 98.6
% of reads
% identity

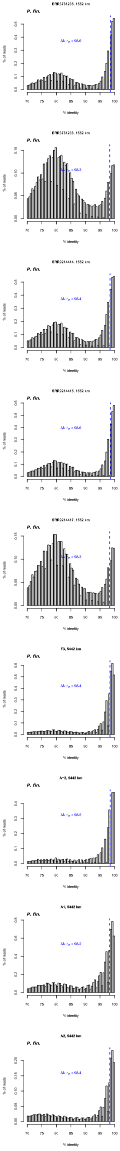

ERR3761235, 1552 km
P. fin.
ANIr$_{95}$ = 98.6
% of reads
% identity
ERR3761238, 1552 km
P. fin.
ANIr$_{95}$ = 98.3
% of reads
% identity
SRR9214414, 1552 km
P. fin.
ANIr$_{95}$ = 98.4
% of reads
% identity
SRR9214415, 1552 km
P. fin.
ANIr$_{95}$ = 98.6
% of reads
% identity
SRR9214417, 1552 km
P. fin.
ANIr$_{95}$ = 98.3
% of reads
% identity
F3, 5442 km
P. fin.
ANIr$_{95}$ = 98.4
% of reads
% identity
A−2, 5442 km
P. fin.
ANIr$_{95}$ = 98.5
% of reads
% identity
A1, 5442 km
P. fin.
ANIr$_{95}$ = 98.2
% of reads
% identity
A2, 5442 km
P. fin.
ANIr$_{95}$ = 98.4
% of reads
% identity

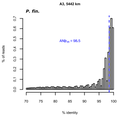
A3, 5442 km
P. fin.
ANIr$_{95}$ = 98.5
% of reads
% identity

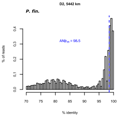
D2, 5442 km
P. fin.
ANIr$_{95}$ = 98.5
% of reads
% identity

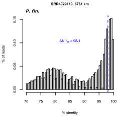
SRR4029110, 6761 km
P. fin.
ANIr$_{95}$ = 98.1
% of reads
% identity

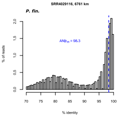
SRR4029116, 6761 km
P. fin.
ANIr$_{95}$ = 98.3
% of reads
% identity

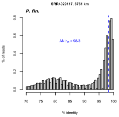
SRR4029117, 6761 km
P. fin.
ANIr$_{95}$ = 98.3
% of reads
% identity

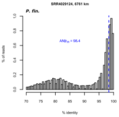
SRR4029124, 6761 km
P. fin.
ANIr$_{95}$ = 98.4
% of reads
% identity

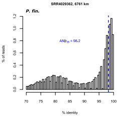
SRR4029362, 6761 km
P. fin.
ANIr$_{95}$ = 98.2
% of reads
% identity

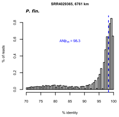
SRR4029365, 6761 km
P. fin.
ANIr$_{95}$ = 98.3
% of reads
% identity

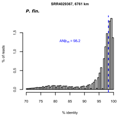
SRR4029367, 6761 km
P. fin.
ANIr$_{95}$ = 98.2
% of reads
% identity

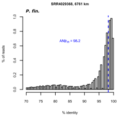
SRR4029368, 6761 km
P. fin.
$ANIr_{95}$ = 98,2
% of reads
% identity

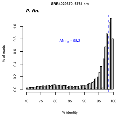
SRR4029370, 6761 km
P. fin.
$ANIr_{95}$ = 98,2
% of reads
% identity

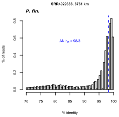
SRR4029386, 6761 km
P. fin.
$ANIr_{95}$ = 98,3
% of reads
% identity

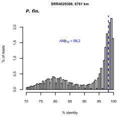
SRR4029388, 6761 km
P. fin.
$ANIr_{95}$ = 98,2
% of reads
% identity

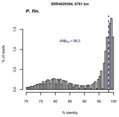
SRR4029394, 6761 km
P. fin.
$ANIr_{95}$ = 98,3
% of reads
% identity

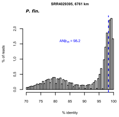
SRR4029395, 6761 km
P. fin.
$ANIr_{95}$ = 98,2
% of reads
% identity

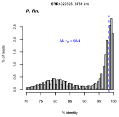
SRR4029396, 6761 km
P. fin.
$ANIr_{95}$ = 98,4
% of reads
% identity

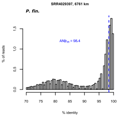
SRR4029397, 6761 km
P. fin.
$ANIr_{95}$ = 98,4
% of reads
% identity

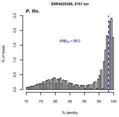
SRR4029398, 6761 km
P. fin.
$ANIr_{95}$ = 98,3
% of reads
% identity

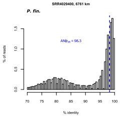
SRR4029400, 6761 km
P. fin.
ANIr95 = 98.3
% of reads
% identity

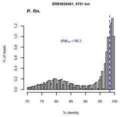
SRR4029401, 6761 km
P. fin.
ANIr95 = 98.3
% of reads
% identity

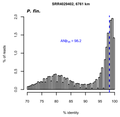
SRR4029402, 6761 km
P. fin.
ANIr95 = 98.2
% of reads
% identity

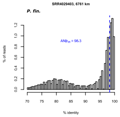
SRR4029403, 6761 km
P. fin.
ANIr95 = 98.3
% of reads
% identity

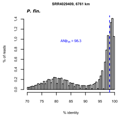
SRR4029409, 6761 km
P. fin.
ANIr95 = 98.3
% of reads
% identity

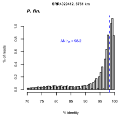
SRR4029412, 6761 km
P. fin.
ANIr95 = 98.2
% of reads
% identity

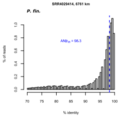
SRR4029414, 6761 km
P. fin.
ANIr95 = 98.3
% of reads
% identity

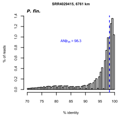
SRR4029415, 6761 km
P. fin.
ANIr95 = 98.3
% of reads
% identity

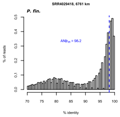
SRR4029418, 6761 km
P. fin.
ANIr95 = 98.2
% of reads
% identity

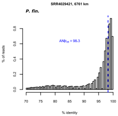
SRR4029421, 6761 km
P. fin.
$ANIr_{95}$ = 98.3
% of reads
% identity

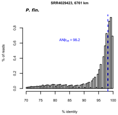
SRR4029423, 6761 km
P. fin.
$ANIr_{95}$ = 98.2
% of reads
% identity

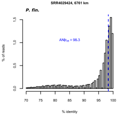
SRR4029424, 6761 km
P. fin.
$ANIr_{95}$ = 98.3
% of reads
% identity
