# Supplementary material for: Geographic population structure and distinct intra-population dynamics of globally abundant freshwater bacteria
Source: ISME J. 2024 Jul 3;18(1):wrae113. doi: 10.1093/ismejo/wrae113 (PMC11283720; doi:10.1093/ismejo/wrae113)
Supplement: SupplTextS1_time_series_dynamics_wrae113 [file suppltexts1_time_series_dynamics_wrae113.pdf]

Supplementary Text S1 – Population abundance and intra-population dynamics

Contrasting strain dynamics as observed between F. sp. in Lake Erken and *P. pan.* in Pond EnzMain (Fig. 6) may be decisively driven by differences in population abundance dynamics. The small variation in the F. sp. population's diversity was accompanied by a similarly small variation in its relative abundance (Suppl. Fig. S10 A). The fraction of based mapped to the F. sp. reference genome was between 0.42 and 0.83% throughout the seven-year time series. Accordingly, the drastic diversity changes in the *P. pan.* population were accompanied by drastic relative abundance changes. In the summer of 2020, the fraction of bases mapped to the *P. pan.* reference genome changed from 0.45% (July) to 6.56% (August) in only 35 days. These bloom-like dynamics are remarkable, given that the genomes of *Polynucleobacter* bacteria are sparsely equipped to respond to environmental changes [1] and *P. pan.* comprises smaller genomes (~ 1.7 Mbp) than most known *Polynucleobacter* species [2,3], which points to a passive lifestyle. In a previous study on a riverine ecosystem, bacterial populations with higher abundance variation showed lower diversity, which was interpreted as a feature of allochthonous, non-persistent community members [4]. While the high dynamics and low diversity of *P. pan.* corroborates the proposed relationship, the consistent detection of the species in Pond EnzMain suggests that it is autochthonous to the habitat. In addition to abundance changes, population size may have also importantly contributed to the striking differences between *P. pan.* and F. sp. in intra-population diversity and dynamics. The surface area of Lake Erken is about 14,000 times larger than that of Pond EnzMain, so we can conclude that the F. sp. population in the former is much larger than the *P. pan.* population in the latter. A larger population is expected to sustain a larger diversity and be less susceptible to changes in diversity [5,6]. Nucleotide diversity in the F. sp. population varied only marginally between 0.46 and 0.47% throughout the seven-year time series, while it changed from 0.26 to 0.12% in the *P. pan.* population within only three months (Suppl. Fig. S10 B). We have suggested a correlation between size and diversity of *P. pan.* populations earlier [7].

References

1. Hahn MW et al. The Passive Yet Successful Way of Planktonic Life: Genomic and Experimental Analysis of the Ecology of a Free-Living Polynucleobacter Population. PLOS ONE 2012;7:e32772., DOI: 10.1371/journal.pone.0032772

2. Hahn MW et al. Fourteen new Polynucleobacter species: P. brandtiae sp. nov., P. kasalickyi sp. nov., P. antarcticus sp. nov., P. arcticus sp. nov., P. tropicus sp. nov., P. bastaniensis sp. nov., P. corsicus sp. nov., P. finlandensis sp. nov., P. ibericus sp. nov., P. hallstattensis sp. nov., P. alcilacus sp. nov., P. nymphae sp. nov., P. paludilacus sp. nov. and P. parvulilacunae sp. nov. Int J Syst Evol Microbiol 2022;72:005408., DOI: 10.1099/ijsem.0.005408

3. Hoetzinger M et al. Polynucleobacter paneuropaeus sp. nov., characterized by six strains isolated from freshwater lakes located along a 3000 km north–south cross-section across Europe. Int J Syst Evol Microbiol 2019;**69**:203–13., DOI: 10.1099/ijsem.0.003130

4. Meziti A et al. Quantifying the changes in genetic diversity within sequence-discrete bacterial populations across a spatial and temporal riverine gradient. ISME J 2019;13:767–79., DOI: 10.1038/s41396-018-0307-6

5. Kimura M. The Neutral Theory of Molecular Evolution. Cambridge: Cambridge University Press, 1983., DOI: 10.1017/CBO9780511623486

6. Star B, Spencer HG. Effects of Genetic Drift and Gene Flow on the Selective Maintenance of Genetic Variation. Genetics 2013;194:235–44., DOI: 10.1534/genetics.113.149781

7. Hoetzinger M et al. Continental-scale gene flow prevents allopatric divergence of pelagic freshwater bacteria. Genome Biol Evol 2021;13., DOI: 10.1093/gbe/evab019
