# Supplementary material for: Geographic population structure and distinct intra-population dynamics of globally abundant freshwater bacteria
Source: ISME J. 2024 Jul 3;18(1):wrae113. doi: 10.1093/ismejo/wrae113 (PMC11283720; doi:10.1093/ismejo/wrae113)
Supplement: SupplFigS12_hclust_group_alleles_wrae113 [file supplfigs12_hclust_group_alleles_wrae113.pdf]

***Polynucleobacter paneuropaeus* - Pond EnzMain**

Split into 9 groups based on Point Biserial Correlation cluster quality

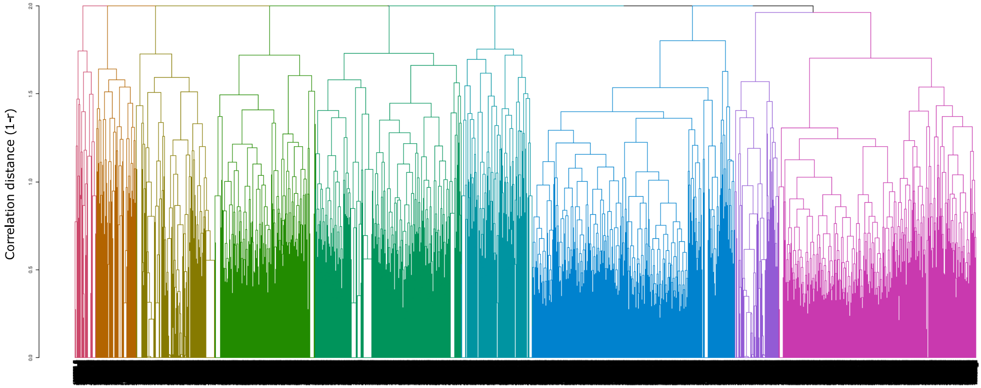

***Ca.* Fonsibacter sp. - Lake Erken**

Split into 11 groups based on Point Biserial Correlation cluster quality

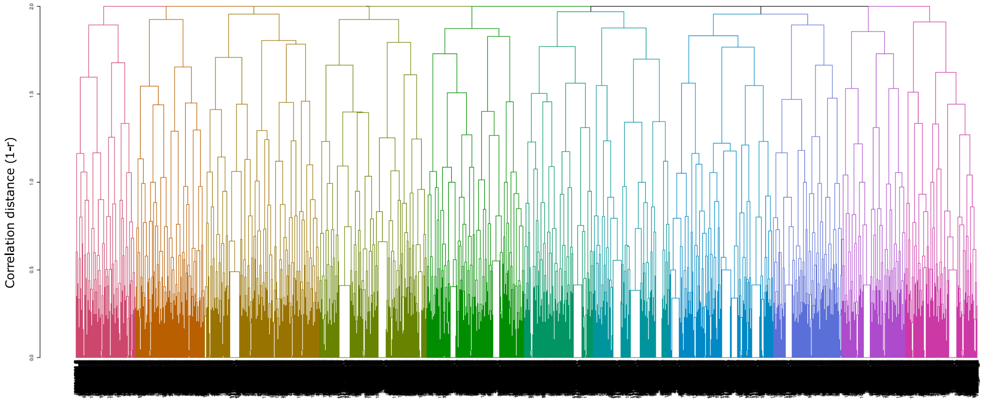

Suppl. Fig. S12.: Hierarchical clustering of alleles based on correlation of allele frequencies across time series of two species in two different habitats. Alleles of the *Polynucleobacter paneuropaeus* population were clustered into nine and those of the *Ca.* Fonsibacter sp. population into eleven groups based on Point Biserial Correlation cluster quality. It is apparent that the groups in *Polynucleobacter paneuropaeus* are more distinct than those in Ca. Fonsibacter sp., pointing at more distinguished genotypes in the former and a more continuous diversity structure in the latter population.
